# Oligonucleotide-Based Therapeutics for STAT3 Targeting in Cancer—Drug Carriers Matter

**DOI:** 10.3390/cancers15235647

**Published:** 2023-11-29

**Authors:** Sara Molenda, Agata Sikorska, Anna Florczak, Patryk Lorenc, Hanna Dams-Kozlowska

**Affiliations:** 1 Department of Cancer Immunology, Poznan University of Medical Sciences, 15 Garbary St., 61-866 Poznan, Poland; sara.molenda@student.ump.edu.pl (S.M.); agata.sikorska@ump.edu.pl (A.S.); annaflorczak@ump.edu.pl (A.F.); patryk.lorenc@student.ump.edu.pl (P.L.); 2 Department of Diagnostics and Cancer Immunology, Greater Poland Cancer Centre, 15 Garbary St., 61-866 Poznan, Poland

**Keywords:** STAT3, oligonucleotide-based therapeutics, nanoparticles, drug delivery systems, cancer therapy

## Abstract

**Simple Summary:**

Activated STAT3 is an essential factor in cancer development; therefore, blocking STAT3 may be of therapeutic benefit in multiple cancer types. Thanks to recent developments in the regulatory function of non-coding nucleic acids, a new branch of nucleic acid-based molecules with significant therapeutic potential has emerged. Therapeutics such as siRNA, shRNA, ASO, and ODN-decoy that target STAT3 found an application in cancer therapies. The main advantage of using nucleic acid-based therapeutics is their high specificity based on the complementarity of the therapeutic sequence with the target. Conversely, oligonucleotide therapeutics struggle with stability, toxicity, sensitivity to nucleases, specificity toward cell type, and cellular uptake, which hamper their applicability. Among the various solutions proposed to overcome these problems, embedding the oligonucleotide in the carrier is an interesting strategy. The review combines the knowledge about STAT3 biology in cancer, the application of anti-STAT3 oligonucleotide therapeutics in cancer therapy, and carriers for their delivery.

**Abstract:**

High expression and phosphorylation of signal transducer and transcription activator 3 (STAT3) are correlated with progression and poor prognosis in various types of cancer. The constitutive activation of STAT3 in cancer affects processes such as cell proliferation, apoptosis, metastasis, angiogenesis, and drug resistance. The importance of STAT3 in cancer makes it a potential therapeutic target. Various methods of directly and indirectly blocking STAT3 activity at different steps of the STAT3 pathway have been investigated. However, the outcome has been limited, mainly by the number of upstream proteins that can reactivate STAT3 or the relatively low specificity of the inhibitors. A new branch of molecules with significant therapeutic potential has emerged thanks to recent developments in the regulatory function of non-coding nucleic acids. Oligonucleotide-based therapeutics can silence target transcripts or edit genes, leading to the modification of gene expression profiles, causing cell death or restoring cell function. Moreover, they can reach untreatable targets, such as transcription factors. This review briefly describes oligonucleotide-based therapeutics that found application to target STAT3 activity in cancer. Additionally, this review comprehensively summarizes how the inhibition of STAT3 activity by nucleic acid-based therapeutics such as siRNA, shRNA, ASO, and ODN-decoy affected the therapy of different types of cancer in preclinical and clinical studies. Moreover, due to some limitations of oligonucleotide-based therapeutics, the importance of carriers that can deliver nucleic acid molecules to affect the STAT3 in cancer cells and cells of the tumor microenvironment (TME) was pointed out. Combining a high specificity of oligonucleotide-based therapeutics toward their targets and functionalized nanoparticles toward cell type can generate very efficient formulations.

## 1. Introduction

The signal transducer and activator of transcription 3 (STAT3) is a member of the STAT protein family [1]. STAT3 has a significant impact on cancers. High STAT3 expression and phosphorylation are associated with poor prognosis for a cancer patient [2,3,4]. STAT3 is constitutively activated in different types of cancer, e.g., breast, lung, prostate, and gastric cancer [5,6,7,8]. Excessive activation of STAT3 affects many tumor-related processes, including cell proliferation, survival, inflammation, invasion, metastasis, and angiogenesis [9]. 

STAT3 belongs to the transcriptional factors (TFs) family and consists of several domains critical for dimerization, transactivation, and DNA binding. The structure of STAT3 can be distinguished an N-terminal domain (NH2), a coiled coil domain (CCD), a DNA-binding domain (DBD), a linker domain (LD), an Src Homology 2 domain (SH2), and a transactivation domain (TAD) [10]. The SH2 domain is required for STAT3 dimerization [11]. 

**Figure 1:**
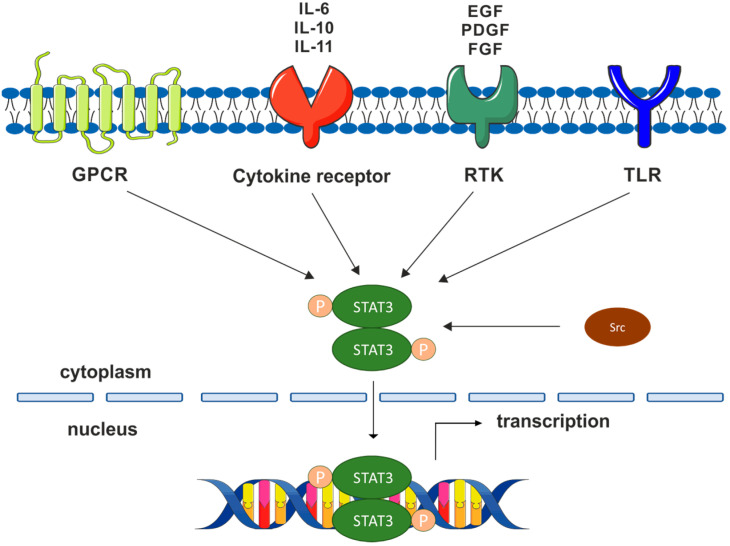
Activation of STAT3 in cancer. The STAT3 pathway is activated by a variety of receptors, such as cytokine receptors, receptor tyrosine kinases (RTKs), G protein-coupled receptors (GPCRs), and toll-like receptors (TLRs). As a result of receptor stimulation, STAT3 is activated through tyrosine phosphorylation. Moreover, STAT3 may be activated by non-receptor tyrosine kinases, including c-Src. Phosphorylated STAT3 dimers translocate to the nucleus, binding to DNA and promoting the expression of specific genes involved in cancer progression. The figure was partly created using the Servier Medical Art Commons Attribution 3.0 Unported Licence.

Canonical activation of STAT3 can be induced by the stimulation of receptor tyrosine kinases (RTK), e.g., epidermal growth factor receptor (EGFR) or cytokine receptors, such as an interleukin-6 (IL-6) receptor [12,13] (Figure 1). IL-6 binds to the IL-6 receptor (IL-6R) and then to the gp130 co-receptor, which leads to the activation of gp130-associated Janus-activated kinase family (JAK) tyrosine kinases [14,15]. The transphosphorylated and fully activated JAKs subsequently phosphorylate multiple tyrosine residues in the cytoplasmic region of gp130 that serve as docking sites for STAT3 [16]. After binding STAT3 through the SH2 domain to phosphorylated tyrosines of gp130, the Tyr705 in the TAD of STAT3 is phosphorylated by JAKs. Phosphorylated STAT3 dimers are then translocated to the nucleus, bind to DNA, and contribute to specific gene expression [17]. Canonical activation of STAT3 can also be caused by non-receptor tyrosine kinases, such as c-Src [18] (Figure 1). Another way to activate STAT3 is through G protein-coupled receptors (GPCRs) [19,20] (Figure 1). However, STAT3 can also affect carcinogenesis by non-canonical pathways. An agent such as MAPK leads to non-canonical phosphorylation of serine 727 (S727), which increases STAT3 transcriptional activity [21]. STAT3 can also be acetylated on lysine 685 (K685) by interacting with p300/CBP histone acetyltransferase protein [22]. Acetylation of K685 enhanced the nuclear localization of STAT3, its ability to bind DNA, and its transactivation activity [23]. The pathway of STAT3 activation in cancer has recently been described in detail in other review papers [16,24,25]. 

**Figure 2:**
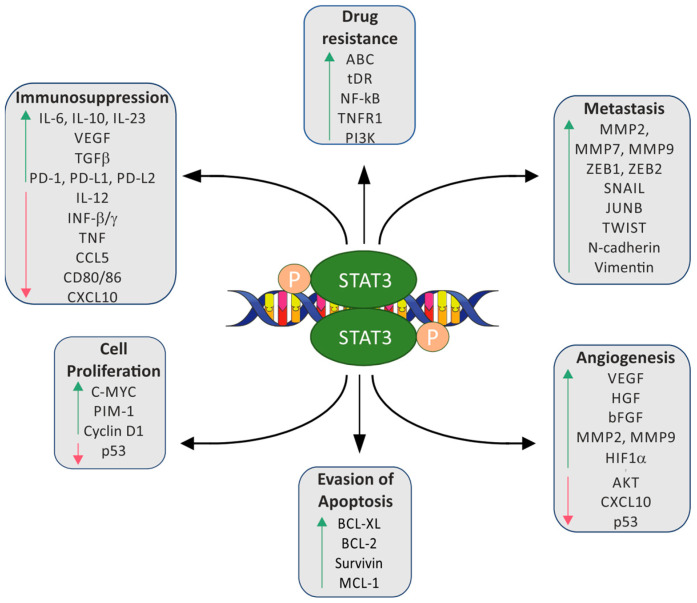
The role of STAT3 in cancer development. STAT3 signaling is involved in many cancer-related processes. Activated STAT3 upregulates the expression of genes related to cell proliferation, anti-apoptosis, drug resistance, and stem-like phenotypes, resulting in the uncontrolled growth of cells, angiogenesis, immune evasion, migration, and invasion. Legend: green arrow—increase, red arrow—decrease. The figure was partly created using the Servier Medical Art Commons Attribution 3.0 Unported Licence.

As mentioned above, STAT3 activation affects many mechanisms important for tumorigenesis (Figure 2). In tumors, the expression of genes related to cell proliferation, such as cyclin D1 (*CCND1*), cyclin B1 (*CCNB1*), and cyclin-dependent kinase 1 (*CDC2*), is increased under the control of activated STAT3 [12,26,27]. Cyclin D1 is one of the primary regulators of the cell cycle that can accelerate cell cycle progression through the G1 phase [28,29]. Cell cycle progression is also stimulated by STAT3-dependent induction of the expression of MYC proto-oncogene (*c-MYC*), serine/threonine protein kinase pim-1 (*PIM-1*) and serine/threonine protein kinase pim-2 (*PIM-2*) [30]. Moreover, constitutively activated STAT3 affects cell survival by regulating the expression of anti-apoptotic molecules of the Bcl family, like extra-large B cell lymphoma (*BCL-xL*), B cell lymphoma 2 (*BCL-2*), and myeloid cell leukemia 1 (*MCL*-1) molecules [31,32,33,34]. 

STAT3 also suppresses apoptosis by stimulating survivin (*BIRC5*) expression [35]. STAT3 may also affect cells’ invasive and migratory properties by regulating the expression of genes such as fibrinogen (*FGA*, *FGG*), chemokine (C-C motif) ligand 2 (*CCL2*), chemokine (C-X-C motif) ligand 2 (*CXCL2*), urokinase-type plasminogen activator (*uPA*), urokinase plasminogen activator surface receptor (*uPAR*), and cathepsins B (*CTSB*) and L (*CASL*) [36]. The expression of E-cadherin (*CDH1*), a tumor suppressor gene that plays a vital role in inhibiting migration and invasion, is inhibited by activated STAT3 [37,38]. Mucin 1 (*MUC1*) can mediate tumor invasion, and its expression depends on STAT3 [39]. Activated STAT3 interacts with paxillin and focal adhesion kinase (*FAK*), which leads to the accumulation of STAT3 in focal adhesion and increases cancer cell invasiveness [40]. Also, phosphorylated STAT3 leads to the upregulation and overproduction of intercellular adhesion molecule 1 (*ICAM 1*), contributing to tumor migration and invasion [41]. However, even unphosphorylated STAT3 may promote tumor cell migration by interacting with the microtubule destabilizing protein—stathmin [42]. 

Metalloproteinases (MMPs) are essential factors in the cancer metastasis process by increasing the motility of cells and regulating epithelial–mesenchymal transition (EMT) [43,44]. Activated STAT3 stimulates the expression of *MMP1*, *MMP2*, and *MMP9* in various types of tumors [45,46,47,48]. Moreover, IL6-induced STAT3 activation stimulates the expression of twist family bHLH transcription factor 1 (*TWIST*), another EMT-related gene [49]. Activated TWIST increases the expression of N-cadherin (*CDH2*) and decreases the expression of E-cadherin [50,51]. On the other hand, cancer cell resistance to cisplatin chemotherapy is related to the STAT3/Snail Family Transcriptional Repressor 1 (*Snail*) axis, which is associated with the development of the EMT phenotype of cells and stem-like properties [52]. Furthermore, overactivated STAT3 is also responsible for forming cancer stem-like cells (CSCs) [53]. 

The vascular endothelial growth factor (VEGF) is one of the most important factors in angiogenesis and vascular development in cancer progression. The VEGF stimulates endothelial cells to invade the matrix and form capillary-like tubules [54]. The VEGF is upregulated by activated STAT3 in many cancers [55,56,57]. Moreover, *VEGF* expression can also be regulated by the hypoxia-inducible factor 1 subunit alpha (HIF-1α) pathway, and hypoxia-induced pSTAT3 accelerates accumulation and prolongs the half-life of the HIF-1α protein [58]. STAT3 may also increase the Warburg effect in neoplastic tissues by upregulating pyruvate kinase M2 (*PKM2*). Higher glucose metabolism in cancer cells increases proliferation and faster tumor development [59].

As mentioned above, activated STAT3 is an essential factor in cancer development; therefore, blocking the activity of STAT3 may be of therapeutic benefit in multiple cancer types. There are many strategies for targeting STAT3 in cancer and other cells of the TME, which can affect the STAT3 signaling pathway at different stages [60]. One of the strategies to target STAT3 is blocking the autocrine molecules that lead to STAT3 activation, such as cytokines and growth factor receptors, using monoclonal antibodies or receptor antagonists. Sant7 is an IL-6 receptor super-antagonist that can efficiently block STAT3 signaling [60,61]. Another way to block STAT3 activation is the inhibition of the activity of upstream tyrosine kinases [62]. AG490 is JAK2-specific inhibitor, and its activity was indicated in the human myeloma U266 model [62]. Cytokines that negatively regulate STAT3 can also be used to target STAT3. SOCS3 is an example of a protein that generates a negative feedback loop to suppress STAT3 signaling [63]. However, all of the mentioned examples have limitations due to the existence of many upstream proteins that can activate STAT3. This means that in such a strategy, the activity of STAT3 cannot be completely inhibited by a single compound [60]. 

Another strategy to downregulate the STAT3 activity directly impacts the STAT3 protein. Stattic is a molecule that by interacting with the SH2 domain, inhibits the dimerization and nuclear translocation of STAT3. It was indicated that stattic sensitized cancer cells for radiation in vitro and in vivo [64]. Another SH2 domain inhibitor, STA-21, reduced breast carcinoma cells’ survival, characterized by STAT3 overactivation [65]. Recently, other SH2 domain inhibitors (323-1 and 323-2) have been developed and lead to the downregulation of STAT3 downstream genes, like the MCL1 apoptosis regulator (*MCL1*) and cyclin D1 (*CCND1*), in DU145 prostate cancer cells [66]. Although the small inhibitors resulted in various anticancer effects, the main obstacle to their application is relatively low specificity. Direct and indirect STAT3 inhibitors have recently been described in detail in other review papers [57,58,59,67], and they are not the subject of this work. However, it should be pointed out that using various nucleic acid-based molecules is another strategy to inhibit STAT3 activity. The main advantage of inhibiting *STAT3* with nucleic acid-based therapeutics is their high specificity based on the complementarity of the therapeutic sequence with the target sequence. 

This review summarizes the types of nucleic acids used to inhibit STAT3 activity, as well as the effect of these molecules on various types of cancer. Moreover, it will be determined the importance of carriers that deliver nucleic acid molecules to affect the STAT3 in cancer cells.

## 2. Therapeutics Based on Nucleic Acids

The role of nucleic acids has recently been significantly expanded beyond coding, storing, and expressing information in every living cell. Moreover, the regulatory function of non-coding nucleic acids has been better understood. Due to these facts, a new branch of molecules with significant therapeutic potential has emerged. Synthetic oligonucleotide therapeutics are more cost-effective and more accessible to develop than small molecule-based drugs. They are specific to a gene or transcript sequence in a complementarity-dependent manner. The task of oligonucleotide therapeutics is to exert a curative effect by silencing targeted transcripts or editing genes. Consequently, nucleic acid-based therapeutics can change gene expression profiles, causing cell death or restoring cell function. Moreover, they may act not only at the nucleic acids level of cells but may also affect the proteins or possess enzymatic activity. They can reach and modulate untreatable targets, such as transcription factors or other intracellular proteins lacking well-defined active sites or enzymatic function [68,69]. Oligonucleotide therapeutics differ in type (DNA, RNA), structure, mechanism of action, function, and exerted effects [70]. Among oligonucleotide therapeutics, which found application in therapies targeting STAT3 function, small interfering RNA (siRNA), short hairpin RNA (shRNA), antisense oligonucleotides (ASOs), and oligodeoxynucleotide decoys (ODN-decoys) can be distinguished.

### 2.1. Molecules of RNAi Pathway

One of the mechanisms to control gene expression is an mRNA degradation pathway called RNA interference (RNAi). RNAi is a natural process that cells use to defend against infecting viruses [71]. Moreover, it plays a pivotal role in maintaining genome integrity through transposable element suppression and the suppression of endogenous genes by promoting DNA methylation, mRNA degradation, or translational repression. The mechanism for silencing the expression of a defined gene was described for the first time in the kingdom of animals in 1998 by Fire A. et al. [72]. The RNAi pathway is complex machinery, and its action depends on enzymes, like double-stranded RNA-specific endoribonucleases (Drosha and Dicer), RNA-induced silencing complex (RISC) and its component endonuclease argonaute 2 (AGO2), and orchestras of proteins that proceed with the maturation of small RNA molecules, finally allowing them to reach specific target sites. Two small RNA molecules are the key elements of the RNAi pathway, i.e., microRNA (miRNA) and short interfering RNA (siRNA).

The development and maturation process of miRNA begins in the nucleus. The miRNAs are transcribed, based on the sequence of their genes, as primary miRNAs (pri-miRNAs). Next, the double-stranded stem-loop pri-miRNA is transformed into pre-miRNA. The microprocessor complex that initiates the maturation process comprises RNase III endonuclease—Drosha. The Drosha enzyme cleaves the pri-miRNA duplex, resulting in double-stranded RNA fragments with a two nucleotide (nt) 3′ overhang called pre-miRNA. Then, pre-miRNA is transported to the cytoplasm via the Exportin 5/RanGTP complex [73]. In the cytoplasm, the Dicer enzyme processes pre-miRNA, resulting in 19–25 nt miRNA [71,73]. This short duplex RNA, the product of Dicer cleavage, consists of two strands: one becomes a guide (antisense) strand and the opposite becomes a passenger (sense) strand. The selection is based on the 5′ end thermostability; the molecule with a less stable 5’ end is recognized as the guide strand. After separation into single strands, the passenger strand is released, while the guide one is incorporated into the RNA-induced silencing complex (RISC). Next, the guide strand anneals to the target mRNA based on partial sequence complementarity [74,75,76,77]. In the miRNA sequence, there is a region called seed. The seed sequence is the minimal region of the miRNA (2–7 nt) at the 5′ end needed to target the mRNA. The miRNA from one family shares the same seed sequence. The effectiveness of the miRNA-mRNA pairing depends on the seed region’s complementarity and the 3′UTR of the target mRNA [78].

The miRNA-dependent regulation of gene expression can be related to the triggering of various effects, including cleaving mRNA, blocking translation, or speeding up deadenylation. The mode of action of a miRNA depends on the complementarity of the miRNA sequence with its target site on the mRNA. The complete complementarity results in binding with AGO2, leading to mRNA degradation by cleavage, while lower complementarity triggers translation inhibition by RISC-binding and blocking ribosomes on mRNA [79]. Another path that leads to mRNA breakdown via an miRNA-dependent manner is accelerating the mRNA deadenylation. The presence of the miRNA-protein complex speeds up the mRNA deadenylation, resulting in faster mRNA degradation [80].

The siRNA molecule can have an endogenous or exogenous origin. Pseudogenes or repetitive elements encode endogenous siRNA and, unlike exogenous siRNA, need nuclear phase processing. Examples of endogenous siRNA are transposons, which occupy mainly centromeres and telomeres positions and play a role in regulating chromatin composition. Exogenous siRNAs originate from invading organisms, like bacteria and viruses [75]. In the cytoplasm, the siRNA molecule follows the same pathway as miRNA and is processed by the Dicer enzyme [81]. siRNA differs from miRNA in length, 21–23 nt, with two nucleotide overhangs at the 3′ end [71]. After Dicer treatment, the RNA duplex interacts with the RISC complex. The activated AGO2 component of the RISC cleaves the passenger strand, forming a guide strand. The complementarity of siRNAs to target mRNA sequences leads to mRNA cleavage by AGO2 [77].

Although siRNA and miRNA are short duplex RNA molecules that target mRNA, the mechanism of their action is distinct. The main difference between siRNA and miRNA is that siRNA silences the expression of one specific mRNA, while miRNA controls the expression of multiple mRNAs. Due to the need for partial complementarity between the seed region of miRNA and the target mRNA, one miRNA molecule can recognize plenty of targets but also increase the number of types of silencing effects. 

#### Artificial RNAi Molecules

Since the RNAi pathway was discovered and its mechanism became better understood, the RNAi molecules started to be used to silence particular genes or modify gene expression patterns deliberately. The era of developing and implementing artificial RNAi-type molecules has begun. Nowadays, there are three RNAi gene silencing platforms: microRNA (miRNA), short interfering RNA (siRNA), and short hairpin RNA (shRNA). Exogenously designed RNAi molecules can be provided directly to the cytoplasm or as a plasmid or viral vector. Due to its mode of action, artificial miRNA therapeutics have not been applied to target STAT3; thus, the review is focused on the characterization of molecules, such as siRNA and shRNA. 

The delivery of synthetic mature siRNA allows for omitting the need for Dicer-dependent cleavage and the possibility of activation of the interferon (IFN) pathway [82]. siRNA must be completely complementary to the mRNA sequence to induce the target cleavage. A commonly used method for the delivery of mature siRNA is an application of lipofectamine, a lipide-based reagent that utilizes an endosomal pathway to enter the cytoplasm [83]. Unfortunately, transfection using lipofectamine is adapted for in vitro introduction of nucleic acids into cells. Thus, many in vivo studies investigating siRNA silencing have used cells treated in vitro with a given siRNA prior to in vivo application. The main difficulties with in vivo siRNA delivery relate to the poor stability of the molecule and off-target effects. 

While siRNA is a relatively short-lived molecule and its effect is transient, a more stable specific gene silencing effect can be achieved using short hairpin RNA (shRNA). The shRNA molecules are exogenously designed and delivered as a plasmid or viral system. After transportation to the nucleus, the shRNA molecule is transcribed and processed in the RNAi pathway, utilizing the endogenous miRNA machinery to generate small RNAs [84]. shRNA consists of 19–22 nt complementary strands linked by a small loop of 4–11 nt to develop a short hairpin structure [85]. An efficient shRNA expression from the plasmid depends on the design of shRNA-transcribed elements. Delivery by virus vectors, like the commonly used lentivirus vectors, results in the integration of vector elements into transcriptionally active chromatin of the host genome. Due to stable integration into the host DNA, shRNA can be constantly expressed. It can exert its effect permanently compared to transient gene knockdown induced by synthetic siRNA [84,85].

These shRNA viral vector constructs carry sequences, including a promoter, encoded shRNAs, and a transcription termination sequence. shRNA in the cell nucleus is usually transcribed by polymerase III (Pol III) or polymerase II (Pol II), depending on the promoter type used. Transcription under the Pol III promoter results in a structure miming pre-miRNA, a hairpin with two nt overhangs at the 3′ end, and a direct substrate for Dicer processing. Thus, the transcription under the control of the promoter processed by Pol III permits the omission of the nuclear form of pri-miRNA. As an shRNA product imitates the structure of the pre-miRNA, it can be transported to the cytoplasm and then utilize the endogenous miRNA processing pathway to make siRNA [84,86].

On the other hand, using Pol II promoters allows for ubiquitous, tissue-specific, or inducible-dependent transcription of shRNA. However, Pol II is less precise than Pol III, resulting in a Drosha-dependent product. The shRNA under the Pol II promoter needs to be designed into the structure of a primary miRNA (pri-miRNA) and requires a more complex maturation process [84,87].

### 2.2. Antisense Oligonucleotides (ASO)

Antisense oligonucleotides are single-stranded, 15–20 nt long, frequently modified synthetic deoxyribonucleic acids [88]. Contrary to most artificial siRNA or shRNA, they are ready-to-use by cells in the cytoplasm; they neither need processing by Drosha nor Dicer enzymes [89]. The chemical modifications can stabilize and protect them against serum nucleases, lower their toxicity, and increase their cellular uptake or binding affinity. They can be delivered to cells’ cytoplasm using various carriers or naturally occurring uptake pathways, like endocytosis [89]. Based on sequence complementarity, they selectively bind to a transcription product. After the target binding, ASOs can cause various effects including alternative splicing, degradation of mature mRNA, or disruption of the translation process. The RNA cleavage pathway is mediated by RNAse H. As ASOs reach the target mRNA and form the RNA-DNA complex, the RNAse H catalyzes target degradation. ASOs are mainly composed of DNA bases or can be chimeras of DNA and RNA bases, named “gapmers”. The hybridization of gapmers to the target has a higher affinity due to the modified RNA-flanking regions. These modifications also provide resistance to degradation by nucleases. The ASOs containing RNA bases may also abolish or promote translation, depending on the binding site [70,88,89,90,91]. 

### 2.3. Oligodeoxynucleotide Decoys (ODN-Decoy)

Another strategy to regulate gene expression is using short double-stranded oligodeoxynucleotide decoys (ODN-decoys). ODN-decoys can be delivered as a plasmid [80], using lipofectamine [92] or cationic solid lipid nanoparticles—SLN [93]. The principle of action of the small nucleic acid molecules known as decoys is based on the imitation of the binding sites for miRNAs or transcription factors. By mimicking miRNAs or transcription factor binding sites, the ODN-decoys compete with their endogenous counterparts for ligand capture [94]. The specific binding of the oligodeoxynucleotide decoy to the transcription factor blocks the binding of that transcription factor to the promoter region, resulting in the inhibition of the transcription process [95]. The miRNA decoys are designed to mimic the specific miRNA family’s seed sequence. They can also be designed to contain several seed sites for a few miRNA families that are separated by spacers [78].

## 3. Delivery of Therapeutics Based on Nucleic Acids

To fulfill their function, oligonucleotide therapeutics must avoid several obstacles in the organism. Premature degradation caused by endonucleases present in physiological fluids and within the extracellular matrix is a major problem for nucleic acid delivery. Moreover, the subsequent barriers are specific cell recognition, sufficient cell binding, and internalization. Premature degradation of oligonucleotide therapeutics in the lysosome compartment can be another hurdle in the cells. The solution to prevent these problems may be a chemical modification of the oligonucleotide therapeutics [96] and/or embedding them in a dedicated carrier [97].

### 3.1. Chemical Modification of Oligonucleotide Therapeutics

Chemical modifications can protect small oligonucleotides from degradation, lower their immunogenic potential, and improve their stability. The standard modification of oligonucleotide bases includes the addition of phosphorothioate (PS), locked and unlocked nucleotides, and substitution of the ribose 2′-OH group [98]. 

The commonly used modification of oligonucleotide therapeutics is the application of phosphorothioate (PS). In PS, the nonbridging oxygen is replaced with a sulfur atom in the oligophosphate backbone. The PS modification of oligo sequences is used to increase its stability. The nonbridging oxygen may also be replaced with more nuclease-resistant and less toxic isoelectronic borane (BH3). However, the BH3-modified RNA is less negatively charged compared to the RNA containing oxygen and sulfur substitutions; thus, it increases the lipophilicity of nucleic acid molecules [99]. 

A frequent modification of the therapeutic ODNs is that they have locked nucleic acids (LNAs) that contain an extra bridge connecting the 2′-oxygen atom and the 4′-carbon atom at various positions. LNA-modified ODNs can activate RNAse H. LNA modification leads to increased molecule stability, melting temperature, and affinity to the complementary sequence of the target [100]. However, the LNA oligonucleotide modification may lead to decreased RNA duplex efficacy [101]. On the other hand, a combination of LNA modification with PS may restore the effectiveness of the modified oligonucleotide [102].

The unlocked nucleic acids (UNAs) lack the RNA ribose ring’s C2′- and C3′- bonds. This modification improves the molecule’s stability. The binding of siRNA to other than the expected sequence region leads to unintentional gene silencing and is related to the miRNA-like effect. The UNA modifications, mainly when included in the seed region, demonstrate a reduction in these off-target effects. This modification also maintains RNA duplex activity [103].

Another type of modification concerns a ribose 2′-OH group in the RNA duplex. It may be substituted by 2′-O-methyl (2′-O-Me), 2′-fluoro (2′-F), and 2′-methoxyethyl (2′-O-MOE), which improve the RNA duplex stability. Such modifications concern the passenger strand, which results in keeping the gene-silencing potency of the RNA duplex.

### 3.2. Carriers Used for the Transportation of Oligonucleotide Therapeutics

As indicated above, oligonucleotide therapeutics delivery is a complex issue. The delivery of oligonucleotide therapeutics in the naked form exposes them to nuclease cleavages. Even chemically modified oligonucleotides that have enhanced resistance to nucleases have a limited duration in circulation. The loading of oligonucleotides into nanoparticles may be a solution to this problem. Conversely, the embedded oligonucleotides into carriers should maintain their availability and properties. Carriers are designed to extend a drug’s circulating half-life, help cross the cellular barriers, and provide targeted delivery, which is essential for in vivo use. The oligonucleotide therapeutics are designed to be specific to mRNA to silence the expression of a given gene. However, they are not specific to the cell type. Therefore, applying functionalized nanoparticles may provide the active delivery of therapeutic nucleic acid into targeted cells, including cancer cells or cells in the tumor microenvironment (TME) [104].

In general, the carriers that can be used for nucleic acid delivery are divided into two groups, namely viral- and non-viral-based systems. Viruses are natural nanocarriers for viral genomes (DNA, RNA); therefore, virus-like particles (VLPs) are the first choice as vehicles for transporting therapeutic oligonucleotides. VLPs derived from different virus species have been engineered for the delivery of nucleic acids, including adenoviruses, Adeno-Associated Viruses (AAVs), lentiviruses, and retroviruses. Depending on the type of VLPs, the nucleic acids can be delivered to replicating/non-replicating cells, their expression can be transient, or the cargo can stably integrate into the host genome, providing its permanent expression. Moreover, VLPs were tested for both in vitro and in vivo delivery of oligonucleotide therapeutics. The viral-based systems as nanocarriers of nucleic acids have been reviewed recently [105,106]. Due to viral-based systems limitations (including safety and immunogenicity), other systems are considered smart, non-viral nucleic acid delivery platforms. Lipids and polymers are the most potent and frequently chosen materials for the production of nanoparticles to replace viral-based systems [97].

To facilitate the efficient delivery of drugs and oligonucleotherapeutics resulting in a response with minimum side effects, solid lipid nanoparticles (SLNs) were made. SLNs are made from biodegradable solid lipids characterized by low toxicity such as glycerides, waxes, fat, oil, triglycerides, and hard fat [107]. These physiological lipids are dispersed and covered by a hydrophilic surfactant. Thus, these cationic nanoparticles bind anionic oligonucleotherapeutics and protect them against degradation by nucleases [93]. 

Others and the most common liposomal nanocarriers are made of phospholipids, which form a spherical vesicle composed of at least one lipid bilayer. Liposomes may incorporate active substances like drugs, peptides, antibodies, and nucleic acids. The newer generations of liposomes are modified with ligands and other polymers [108]. The ligands can provide the targeting delivery by recognizing specific receptors on the cell surface and then facilitating their internalization. Functionalization by polymers or molecules like poly(ethylene glycol) PEG can stabilize the structure of liposomes, which is essential for in vivo circulation [109]. Another type of liposomal carrier is lipoplexes, i.e., cationic liposomes. Lipoplexe carriers are capable of binding oligonucleotides due to electrostatic interaction. Moreover, the cellular uptake of positively charged lipoplexes may be high, corresponding to the need for a lower drug dose. Furthermore, lipoplexes exhibit relatively low cytotoxicity at the dose needed to deliver a drug to trigger an outcome [110,111,112]. 

The other nanoparticles belonging to the group of lipids are stable nucleic acid–lipid particles (SNALPs). SNALPs are composed of fusogenic and cationic lipids that can be internalized by cells via an endosomal pathway [97]. They demonstrated a high nucleic acid encapsulation efficiency and prolonged siRNA half-life even after systemic delivery [113,114]. The binding of oligonucleotides to SNALPs reduced oligonucleotide-dependent toxicity by lowering the required dose of oligonucleotide-based therapeutics [113]. Numerous studies on therapeutic oligonucleotide delivery efficiency, toxicity, and half-life activity in mice and non-human primate models indicated that SNALPs are promising nanoparticles [115,116,117,118]. 

A variety of polymers, such as polyethyleneimine (PEI), and polysaccharides, such as cyclodextrin (CD) and chitosan, have also been shown to be capable of carrying oligonucleotide-based therapeutic agents [119]. These cationic carriers are abundant in positively charged groups, such as amines and amides, and can form various shapes, such as linear, branched, or dendritic structures [120,121]. 

Polyethylenimine (PEI) is a cationic polymer carrier containing the amine group and an aliphatic carbon spacer. It can be obtained in the shape of branched and linear forms. Branched PEI is more reactive and forms small complexes with the oligonucleotide, approximately 100 nm in diameter. The small size of the PEI-oligonucleotide complex protects it against removal from the blood by the complement system [122]. The linear PEI demonstrates reduced toxicity and higher transfection potential than the branched one. Various modifications of PEI, such as acid modification or PEGylation, can enhance oligonucleotide uptake by reducing particle dose and decreasing PEI toxicity [123,124,125].

Chitosan is a linear polysaccharide obtained by the alkaline N-deacetylation of chitin. It is biodegradable, biocompatible, and demonstrates low toxicity with a high affinity to oligonucleotides. It is soluble in acid but not in neutral pH [126]. This property affects the different behavior of the material depending on the pH and reduces the transfection efficiency [127]. However, these characteristics can be used as an advantage and utilized for generating a pH-dependent carrier for drug delivery to an acidic tumor microenvironment [128].

Other studied polymers for the efficient and safe delivery of oligonucleotide-based therapeutics are polyesters, including poly(lactic acid) (PLA), poly(glycolic acid) (PGA), and copolymer poly(lactic-co-glycolic acid) (PLGA). The advantage of PLGA, among other polyesters, is its low toxicity at the expense of poor encapsulation efficiency of oligonucleotide-based therapeutics. On the other hand, drug encapsulation yield, delivery efficacy, and cellular uptake can be improved by mixing PLGA with cationic polymers, like PEI or chitosan [97,125,129,130]. 

Inorganic nanoparticles are metal oxide or metallic composition particles. Among common inorganic nanomaterials, such as quantum dots (QDs), gold nanoparticles (AuNPs), silver nanoparticles (AgNPs), carbon nanotubes (CNTs), and mesoporous silica nanoparticles (MSNs), AuNPs are the most often used for oligonucleotide therapeutics delivery [121,131]. Nucleic acids covalently attached to AuNPs are protected against nucleases and demonstrate increased cellular uptake, which can be further enhanced by the formation of an AuNP-nucleic acid complex with PEI [132]. Moreover, except for the drug delivery potential, AuNPs have unique optics and electronic and surface plasmon resonance characteristics, which can be used for photothermal therapy. Additionally, the nuclear localization of AuNPs causes DNA damage via physical dose enhancement by the short-range low-energy electrons [133]. AuNPs are easy for preparation, modification, and functionalization with active ligands via Au–S chemical bonds [134].

## 4. Oligonucleotide-Based Therapeutics Targeting STAT3 Delivered into Cancer Cells in a Naked Form

**Figure 3:**
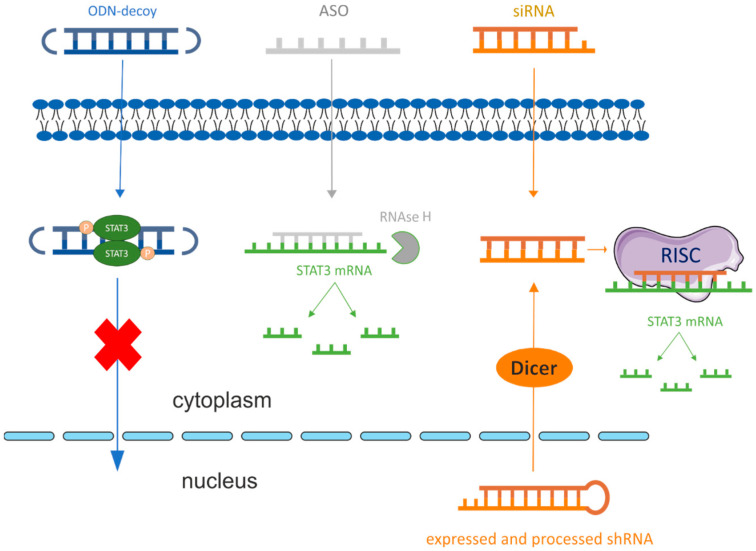
STAT3-targeting strategies with oligonucleotide-based therapeutics. Oligonucleotide therapeutics used in STAT3-inhibiting therapies are oligodeoxynucleotide decoys (ODN-decoys), antisense oligonucleotides (ASOs), small interfering RNA (siRNA), and short hairpin RNA (shRNA). ODN-decoys are double-stranded oligodeoxynucleotides that mimic the binding sequence of STAT3. ASOs, the single-stranded deoxyribonucleic acids, selectively bind to a transcription product, leading to their degradation by the RNase H pathway. RNA interference (RNAi) molecules, siRNA and shRNA, associate with the RNA-induced silencing complex (RISC) and anneal to the target mRNA and lead to its degradation and thus gene expression silencing. The figure was partly created using the Servier Medical Art Commons Attribution 3.0 Unported Licence.

Various types of molecules based on nucleic acid were used to inhibit the activity of STAT3 in cancer. Among the most commonly used were siRNA, shRNA, ASO, and ODNs-decoy (Figure 3). Initially, scientists analyzed the activity of oligonucleotide-based therapeutics in naked form, but the use of dedicated carriers was applied over time. However, applying nucleic acid therapeutics in a naked form provided much information about their capabilities. Moreover, due to the increased number of nucleic acid sequence modifications, their application can be expanded. The data describing the application of nucleic acid-based therapeutics delivered in the naked form to inhibit STAT3 in cancer are summarized in Table 1.

### 4.1. siRNA-Based Therapeutics 

The silencing of *STAT3* in tumors with naked siRNA was a frequently used method. In vitro,, the silencing of *STAT3* in colorectal cancer (CRC) induced a drop in *BIRC5* mRNA level and increased *TP53* and caspase-3 (*CASP3*) [135]. Moreover, the proliferation of HCT-116 and SW480 colorectal cancer cells decreased after *STAT3* silencing. Furthermore, these cells had a significantly higher apoptosis rate than the control non-treated ones. In mice, a tumor formed of HCT-116 cells after silencing *STAT3* grew slower than the control group. The tumor sample treated with siRNA *STAT3* was characterized by an increased level of p53 and caspase-3 and a decreased level of survivin proteins [135]. 

Comprehensive *STAT3* silencing studies in gastric cancer have revealed that STAT3 is an important factor in cancer progression [136]. In vitro studies silencing *STAT3* by specific siRNA reduced proliferation and increased the percentage of apoptosis of human SGC-7901 gastric cancer cells. Furthermore, it led to the arrest of the cell cycle in the G1 phase. Western blot analysis revealed that the level of STAT3-dependent proteins, e.g., cyclin-D1, survivin, and BCL-2, was significantly lower in the treated group than in the control groups. They also observed a reduced proliferation rate of tumor cells in mice treated with *STAT3* siRNA [136].

Studies conducted by Li et al. revealed that the silencing of STAT3 in the hepatocellular carcinoma model reduced glycolysis-related gene—Hexokinase 2 (*HK2*)—expression observed at the mRNA and protein levels. Furthermore, transfection with *STAT3* siRNA reduced glucose consumption and lactate production in hepatocarcinoma HepG2 and Hep3B cells, decelerating glycolysis, thereby reducing the Warburg effect and cell proliferation [137]. The study conducted by Zhang and colleagues demonstrated a higher apoptosis rate of cells treated with *STAT3* siRNA compared to control groups in hepatocarcinoma Bel-7402 cells [138]. A higher cell apoptosis ratio might be related to the disruption of mitochondria. Furthermore, Western blot analyses showed an increased level of cleaved caspase-3 (17, 19 kDa) in modified cells; however, there was no difference in the protein level of full-length caspase-3 [138]. 

In lung cancer, Wang et al. showed a decreased proliferation of Lewis lung cancer cells treated with *STAT3* siRNA [139]. Moreover, the rate of the apoptotic cells was increased by 19% in the treated group compared with the control groups [139]. In vitro, research conducted on HCC827, HCC827ER, and H1975 lung cancer cells using a combination treatment of siRNA *STAT3* with erlotinib (epidermal growth factor receptor tyrosine kinase inhibitor) revealed an association of erlotinib resistance with *STAT3* activation [140]. Treatment with erlotinib induced apoptosis of lung cancer HCC827, HCC827ER, and H1975 cells, but using both erlotinib with *STAT3* siRNA significantly enhanced this effect [140]. 

In vitro studies based on the K562 leukemia cell line showed the influence of *STAT3* silencing on several cellular processes [141]. Cells treated with *STAT3* siRNA had a lower proliferation rate than control non-treated cells. Apoptosis analysis revealed the highest ratio of apoptotic cells in *STAT3* siRNA-treated cells. Moreover, cell cycle analysis indicated the G1 to S phase arrest in these cells [141]. Studies conducted by Xiao et al. revealed that STAT3 and C-C motif chemokine ligand 4 (CCL4) were involved in the progression of diffuse large B cell lymphoma (DLBCL) [142]. The expression of *CCL4* in DLBCL cells treated with *STAT3* siRNA decreased, which affected the Wnt/β-catenin pathway. *STAT3* silencing reduced the proliferation of SU-DHL-8 and OCI-LY1 DLBCL cells, which could be partially restored by *CCL4* overexpression. Moreover, the in vitro study indicated that cells with lower levels of STAT3 had reduced potential for migration and invasion. Cell cycle analysis revealed an increased ratio of the G0/G1 phase and a significantly reduced amount of S phase in cells treated with *STAT3* siRNA. They also showed that all described changes in *STAT3* silencing can be reversed by *CCL4* overexpression [142]. Zhang et al. investigated CpG-siSTAT3 therapeutics in leukemia and myeloma models [143]. The CpG-siSTAT3 consists of oligodeoxynucleotide CpG that binds to TLR9 and *STAT3* siRNA. The expression of *STAT3* after CpG-*STAT3* therapeutics treatment was lower in KMS-11 myeloma cells. Moreover, the growth of MV4–11 leukemia and KMS-11 myeloma tumors was inhibited after intratumorally CpG-*STAT3* administration [143]. 

Immunohistochemistry and protein analyses of STAT3 in ovarian cancer showed that the level of STAT3 significantly increased in ovarian cancer tissues and cell lines [144]. Therefore, Zheng’s group investigated the influence of *STAT3* silencing on this type of cancer. Western blot and RT-PCR analysis showed that the inhibition of STAT3 in SKOV3 and OVCAR3 ovarian cancer cells reduced the expression of *CCND1*, *BIRC5*, and *VEGF*. Cell proliferation of *STAT3* siRNA-treated cells was significantly lower than the control cells. Moreover, further study showed an increased number of apoptotic cells among *STAT3* siRNA-treated cells. The xenograft model of the ovarian tumor made of OVCAR3 cells with *STAT3* silencing grew slower than the control cells without treatment [144]. It was indicated that ovarian cancer might develop chemoresistance to cisplatin through the anti-apoptotic effect, which was correlated with a high level of pSTAT3 protein [145]. Cisplatin-resistant C13K and SKOV3 ovarian cancer cell lines treated with *STAT3* siRNA and cisplatin have an increased ratio of dead cells compared to cells receiving cisplatin alone. Furthermore, cisplatin-sensitive OV2008 and A2780 ovarian cancer cell lines treated with IL-6, which activates STAT3, showed increased cisplatin resistance. Silencing *STAT3* in these cells by siRNA also reduced the IL-6-induced cisplatin resistance. These data indicated that the silencing of *STAT3* can be used to treat chemoresistant ovarian cancer [145].

Analysis of STAT3 and STAT3 tyrosine 705 phosphorylation in human astrocytes and astrocytoma cell lines showed that STAT3 was overexpressed and overactivated in cancer cells [146]. *STAT3* siRNA-treated A172 and T98G astrocytoma cells had different morphology, e.g., cells were smaller and more rounded than wild correspondent types. Astrocytoma cells’ viability significantly decreased compared with normal astrocytes after being treated with *STAT3* siRNA. A higher percentage of apoptotic nuclei, a higher level of caspase-3 protein, and a higher percentage of apoptotic cells among *STAT3*-silenced astrocytoma cells suggested that STAT3 plays an important role in inhibiting apoptosis. Moreover, a decreased level of survivin and Bcl-xL protein by Western blot after siRNA-*STAT3* application was reported, which negatively correlated with cell survival [146]. In retinoblastoma ARPE-19, HRMECs, Y79 cells, and the silencing of *STAT3* by siRNA reduced cell proliferation and the expression of STAT3-related genes, such as *BCL2*, *BCL2L1*, *BIRC5*, *MMP9*, *VEGFA*, *CCND1*, cyclin-dependent kinase inhibitor 1A (*CDKN1A*), and *MYC* [147]. Moreover, in vivo, the orthotopic tumors of Y79 cells treated intravitreally with *STAT3* siRNA did not form externally visible tumors. The histologic examination of the tumor sample also confirmed a reduced amount of retinoblastoma tumor cells in the vitreous cavity between the lens and the retina in the *STAT3* siRNA-treated group compared with the control [147].

The silencing of *STAT3* in oral squamous cell carcinoma (SCC) was correlated with the activation of the IFNγ pathway [148]. Transfection with *STAT3* siRNA reduced the proliferation of GFP-SAS, HSC-4, HSC-3, and KB SCC cell lines. The decreased expression of *STAT3* was also correlated with the lower expression of *CCND1* and *VEGF*. In contrast, the expression of *MMP10* did not change after the silencing of *STAT3* in oral SCC [148]. The silencing of *STAT3* in human laryngeal SCC was correlated with enhanced radiosensitivity of these cells compared with the control. Hep-2 cells treated with *STAT3* siRNA and irradiation had increased apoptosis and a lower proliferation rate than those treated only with irradiation. Moreover, STAT3-dependent downstream proteins, such as BCL-2, VEGF, and p53, also decreased in double-treated cells [149]. The inhibition of STAT3 in the hypopharyngeal cells might prevent carcinogenesis caused by acidic bile (BA) [150]. Vageli et al. indicated that applying *STAT3* siRNA into normal human hypopharyngeal cells (HCs) reversed the “mRNA oncogenic phenotype” caused by acidic bile. The transfected cells had lower expressions of *IL6*, *TNF-α*, *BCL2*, *RELA*(*P65*), *STAT3*, REL proto-oncogene, NF-kB subunit (*REL*), Wnt family member 5A (*WNT5A*), and *EGFR* compared to cells treated with acidic bile alone. Furthermore, these BA-induced cells of an oncogenic nature, after treatment with *STAT3* siRNA, had a lower survival rate than the control BA-induced cells [150]. 

STAT3 is also an essential apoptotic factor in breast cancer [151]. Kunigal et al. showed that the silencing of *STAT3* could stimulate the apoptosis of MDA-MB-231 breast cancer cells, which correlated with the inhibition of the expression of survival genes, e.g., *BCL-xL* and *BIRC5*. On the contrary, the expression of the Fas cell surface death receptor (*FAS*) and Fas ligand (*FAS-L*) and their downstream molecule—Fas-associated via death domain (*FADD*)—was significantly higher in *STAT3*-siRNA treated cells. Further analyses of proapoptotic factors showed that the silencing of *STAT3* increased the cleavage of the effector caspase-3, initiator caspases 8 and 9, and the cleavage of poly (ADP-ribose) polymerase-1 (PARP1). Moreover, analysis of the cytosolic fraction also showed increased signalization for cytochrome C and Diablo IAP-binding mitochondrial protein (SMAC), which participate in apoptosome formation. Further, in vivo studies revealed that the growth of *STAT3* siRNA transfected MDA-MB-231 breast cancer cells was significantly lower than in control mice and was associated with an increased number of apoptotic cells in the tumor site. In addition, the protein analysis showed a decreased level of STAT3 and BCL-xL, an increase in FAS and FAS-L, and an increase in the amount of cleaved caspase-3. The in vitro and in vivo data strongly indicated that activated STAT3 is a critical anti-apoptotic factor in breast cancer [151]. Another study using RCC4 renal clear cell carcinoma and MDA-MB-231 breast cancer cells indicated that *STAT3* siRNA decreased the hypoxia-induced expression of *HIF1*-dependent genes such as pyruvate dehydrogenase kinase 1 (*PDK1*), angiopoietin-like 4 (*ANGPTL4*), carbonic anhydrase 9 (*CA9*), and *HIF1/2*-dependent *VEGF* [152]. However, the silencing of *STAT3* did not affect *HIF2*-specific targets, such as G protein-coupled receptor 157 (*GPR157*) and placenta-associated 8 (*PLAC8*). Further functional experiments showed that the silencing of *STAT3* reduced the proliferation, migration, and clonogenic survival of MDA-MB-231 breast cancer cells under hypoxic stimulation. The proliferation and migration of RCC4 cells were also decreased under the normoxic condition, but their clonogenic survival was enhanced by *STAT3* siRNA [152]. ChIP-seq and RNA-seq analysis of the Triple Negative Breast Cancer (TNBC) conducted by McDaniel et al. revealed novel gene signatures directly regulated by STAT3 [82]. Further in vitro analysis showed that the treatment of HCC70 and MDA-MB-231 TNBC cells with *STAT3* siRNA reduced the invasive potential of these cells compared with the non-target siRNA control but did not affect the cell viability, as was predicted by ChIP analyses [82]. 

Constitutive expression of *STAT3* in prostate cancer was correlated with enhanced tumor growth [153]. Lee et al. developed siRNA targeting *STAT3* and applied it in a prostate cancer model. DU145 and LN-17 prostate cancer cell lines treated with *STAT3* siRNA were characterized by decreased proliferation compared with cells treated with control siRNA. Their growth inhibition was *STAT3* siRNA dose–dependent. However, the proliferation in the STAT3-negative PC-3 prostate cancer cell line did not change after *STAT3* siRNA application. Further study indicated that *STAT3* siRNA induced the apoptosis of DU145 and LN-17 cells, while the apoptosis rate of the PC-3 cell line was not modified. The Prostate-Specific Antigen (PSA) level positively correlates with the clinical stages of prostate cancer. Lee et al. showed that the silencing of STAT3 decreased PSA expression in LN-17 prostate cancer cells [153]. Moreover, prostate cancer cells showed the presence of the TLR9 receptor, which can bind to the CpG molecule [154]. Moreira et al. indicated that CpG-*STAT3* siRNA could be internalized by TLR9-positive PC3 and DU145 prostate cancer cells. The intratumoral administration of CpG *STAT3* siRNA inhibited the growth of PC3 and DU145 tumors in vivo. Moreover, colony-forming assays showed that CpG-*STAT3* siRNA reduced the clonogenic potential of tumor cells [154]. 

### 4.2. shRNA-Based Therapeutics 

Another frequently used method of *STAT3* silencing in cancer cells is the application of naked shRNA in a plasmid form. This molecule is transported to the nucleus and then proceeds in the RNAi pathway to siRNA through an endogenous process. By assimilation into the endogenous pathway, its application requires a lower dose and is more efficient than artificial, ready-to-use, naked siRNA.

The transfection of CAOV3 ovarian cancer cells with *STAT3* shRNA plasmid resulted in a reduction in STAT3 and phosphorylated STAT3 protein amount [155]. *STAT3* shRNA inhibited the proliferation and anchorage-independent growth of CAOV3 cells. Moreover, cells treated with *STAT3* shRNA vectors were characterized by increased apoptosis compared with cells treated with shRNA-scrambled vectors. Furthermore, the BCL-xL and cyclin D1 protein levels decreased in cells upon *STAT3* silencing. Further, in vivo study using a tumor model made of CAOV3 ovarian cancer cells with silenced *STAT3* indicated that the formed tumor was significantly smaller than the control cells [155]. Studies conducted by Wen et al. using the xenograft ovarian cancer model made of SKOV3 cells also revealed that silencing *STAT3* by shRNA reduced metastasis [156]. Moreover, *STAT3* shRNA decreased the expression of *IL-6* in ovarian cancer cells in vitro and the ovarian xenograft tumor model [156].

Silencing *STAT3* by shRNA in lung cancer cell lines A549 and SPC-A1 suppressed STAT3 on mRNA and protein levels [157]. In vitro study indicated that lung cancer cells treated with *STAT3* shRNA and cisplatin had decreased cell viability and the ability for colony formation. Moreover, the silencing of *STAT3* by shRNA in combination with cisplatin increased apoptosis and the caspase-3 activity in lung cancer cells compared with treatment with cisplatin alone [157]. Yin et al. investigated the combined effect of *STAT3* silencing by shRNA and radiotherapy on lung cancer cells [158]. The number of surviving SK-MES-1 and A549 lung cancer cells treated with *STAT3* shRNA and irradiation was lower than the untreated control cells. Moreover, the ratio of apoptotic cells increased in the shRNA *STAT3*-treated group. Further in vivo experiments of tumors made of SK-MES-1 and A549 cells transfected with *STAT3* shRNA indicated that these cells were more radiosensitive than the control untransfected cells or cells transfected with nonspecific shRNA [158].

Li et al. showed that the silencing of *STAT3* by shRNA in laryngeal squamous cell carcinoma enhanced tumor radiosensitivity in vivo [159]. A reduction in laryngeal squamous tumor growth in the plasmid sh*STAT3* treated mice or irradiated (IR) group alone was shown. However, the most significant tumor growth reduction was observed in the mice after simultaneous sh*STAT3* and radiation application. Moreover, in tumors treated with sh*STAT3* and IR, the level of p53, BCL-2, and VEGF proteins was reduced, as well as neovasculature, and an increase in the apoptosis of laryngeal squamous carcinoma cells was observed compared with the control groups [159]. 

*STAT3* shRNA can reduce specific and sustained EGFR-dependent activation of STAT3 in the HN5 head and neck tumor cell line [160]. Cells treated with *STAT3* shRNA were characterized by reduced growth, and it was suggested that this might be caused by a restoration of transforming growth factor β (TGF-β) cytostatic abilities. Moreover, the growth of tumors made of HN5 cells with *STAT3* silencing was also reduced in the xenografts model compared with untreated tumors [160]. 

GRIM-19 is a growth-suppressive protein that can bind to the *STAT3* gene, inhibiting the transcription of *STAT3* [161]. The simultaneous silencing of *STAT3* using shRNA and the overexpression of *GRIM-19* synergistically increased the apoptosis of PC-3M prostate cancer cells. Further studies revealed that the expression of anti-apoptotic genes *BCL-2*, *CCND1*, *c-MYC*, and *VEGF* was reduced in cells treated with a plasmid that co-expressed *GRIM-19* and *STAT3* shRNA (GRIM-19-Si-Stat3). GRIM-19-Si-Stat3 inhibited tumor growth and increased the apoptosis of cancer cells in vivo in a prostate cancer xenograft model. The combined therapy was more effective than applying these agents separately [161]. 

There was an increased STAT3 activation in HER2-positive breast cancer cells that was related to the stem-like potential of these cells [162]. The silencing of *STAT3* by shRNA in HER2 overexpressing breast cancer cells resulted in a reduction in CD44-positive cells and the downregulation of the expression of stem cell markers, such as Octamer-binding transcription factor (*OCT-4*) and SRY (sex determining region Y)-box 2 (*SOX-2*). The silencing of *STAT3* in MCF7-HER2 breast cancer cells also reduced their ability to form tumorspheres caused by the overexpression of Erb-b2 receptor tyrosine kinase 2 (*ERBB2*) that encodes HER2 [162]. 

Studies conducted by Vang et al. demonstrated that *STAT3* shRNA-mediated silencing inhibited the proliferation, promoted apoptosis, and cell cycle arrest of HepG2.2.15 HBV+ hepatocellular carcinoma cells [163]. Moreover, *STAT3* silencing reduced the expression of angiogenesis-related genes such as *VEGF*, *MMP-9*, and *TGF-β* in hepatocellular carcinoma cells. In vivo studies showed that mice with hepatocellular tumors intratumorally injected with lipofectamine 2000-*STAT3* shRNA complexes were smaller compared with control tumors [163]. 

*STAT3* shRNA inhibited proliferation and downregulated the expression of *c-MYC*, *CCND1*, and *BRIC5* in vitro and in vivo in the SCC-3 lymphoma cells. Moreover, the expression of the tumor suppressor gene Latexin (*LXN*) was restored after *STAT3* silencing, which might be related to observed tumor regression [164]. 

### 4.3. ASO-Based Therapeutics 

ASO is a tool that can execute various effects related to gene expression. Due to the application of oligonucleotide chemistry and various modifications, ASO safety, selectivity, and potency have increased. STAT3 ASO also found applicability to treat cancer. 

STAT3 silencing using STAT3 ASO in HCCLM3, SNU423, and Huh7 hepatocellular carcinoma cells reduced cell proliferation, survival, and migration [165]. The in vitro expression of VEGF, MMP2, MMP9, BRIC5, AKT-1, BCL-xL, c-MYC, and CCND1 decreased in HCCLM3 cells after the application of STAT3 ASO. Moreover, Fas and TNFα expression increased in treated cells compared with the control. Further in vivo studies of an HCCLM3 hepatocellular carcinoma mouse model showed that the level of circulating VEGF decreased after STAT3 ASO treatment. Moreover, STAT3 ASO application inhibited angiogenesis, reduced tumor metastasis, and extended the survival of hepatocellular carcinoma-bearing mice compared with the control groups [165]. 

Research conducted by Hong and colleagues using the lymphoma model was based on the application of AZD9150, a next-generation antisense oligonucleotide inhibitor of STAT3 [89]. They showed that in vitro AZD9150 inhibited SUP-M2 and KARPAS299 lymphoma cell proliferation by inducing apoptosis through a pathway associated with PARP1 cleavage or the activation of caspase-3. Moreover, AZD9150 treatment decreased the expression of STAT3-dependent genes such as MCL1, c-MYC, B cell lymphoma 6 transcription factor (BCL6), CCND1, BRIC5, and IL-2Rα. The systemic delivery of AZD9150 to mice bearing SUP-M2 tumors lowered the level of STAT3 protein in the tumor samples compared with the control. Moreover, after the application of AZD9150 in vivo, tumor growth was inhibited [89]. 

### 4.4. ODN-Decoy-Based Therapeutics 

Another strategy for targeting STAT3 using nucleic acid-based therapeutics in cancer is the application of ODN-decoy molecules. The double-stranded ODN-decoy may mimic the specific site to which a transcription factor binds. This type of inhibition of transcription factors is very specific and efficient [166]. 

In the pulmonary giant cell carcinoma cell line (PG), the application of the STAT3 ODN-decoy was responsible for inhibiting cell proliferation and the downregulation of the expression of genes related to cell cycle and apoptosis such as *MCL-1*, *CCND1*, *BCL-xl*, and *BRIC5* [167]. Njahta et al. found that a cyclic STAT3 ODN-decoy (CS3D) inhibited colony formation and induced the apoptosis of Non—Small-Cell Lung Cancer (NSCLC) cells [168]. The STAT3-dependent expression of *c-MYC* and *BCL-xL* also decreased in NSCLC cells compared with the control group. In vivo studies indicated that intravenous injection of CS3D resulted in tumor growth inhibition in the NSCLC mouse model. The analysis of residual tumors showed decreased *c-MYC* expression in CS3D-treated samples [168]. 

In the study conducted by Rahmati and colleagues, the STAT3 ODN-decoy was used for breast cancer therapy [92]. Treatment of MDA-MB-231 cells with the STAT3 ODN-decoy reduced cell proliferation, mammospheroid formation, arrested cell cycle at the G0/G1 phase, and increased cell apoptosis compared with the control cells. In vivo studies revealed that the STAT3 ODN-decoy inhibited metastatic properties, like invasion and migration. In this model, the oligonucleotide-based therapeutic-treated cells were characterized by a decrease in CD44 and an increase in the amount of CD24 protein on the cell surface, which may be related to the differentiation of breast cancer cells related to losing stem-like phenotypes. Moreover, *BCL-xL*, *CCND1*, and *c-MYC* expression decreased in treated cells [92]. Another in vitro study using MDA-MB-231 breast cancer cells resistant to chemo- and radiotherapy has shown that the STAT3 ODN-decoy could restore cell sensitivity to treatment [95]. Blocking STAT3 activity significantly improved the effectiveness of individual chemo- and radiotherapy; however, combined therapy with radiation, methotrexate, and the STAT3 ODN-decoy resulted in the best results. All three treatments significantly reduced cell viability, arrested the cell cycle, and induced cell apoptosis in vitro. The combination also decreased breast cancer cell invasion and migration potential [95].

The treatment of SKOV3 and OVCAR3 ovarian cancer cells with the STAT3 ODN-decoy led to decreased invasive cancer potential and increased cell sensitivity to paclitaxel [169]. Moreover, the STAT3 ODN-decoy caused a reduction in the level of the extracellular matrix metalloproteinase inducer (EMMPRIN), a marker of ovarian cancer metastasis, P-glycoprotein (P-GP), and phosphorylated RAC-alpha serine/threonine protein kinase (p-AKT) proteins, which are responsible for chemoresistance in ovarian cancer cells [169]. Other in vitro studies based on the use of the STAT3 ODN-decoy in OVCAR3 and SKOV3 ovarian cancer have shown that it inhibited cell proliferation, led to cell cycle arrest, and inducted cell apoptosis [170]. The effect of the STAT3 ODN-decoy on the inhibition of ovarian cancer growth and the induction of cell apoptosis has also been confirmed in in vivo studies. In addition, the application of the STAT3 ODN-decoy in tumors made of SKOV3 cells resulted in a significant reduction in MMP2, MMP9, and BCL-2 proteins compared with the control tumors. These changes in the protein levels might cause a reduction in metastasis and an induction of apoptosis of ovarian cancer cells [171]. 

In vitro, the application of the STAT3 ODN-decoy to treat the malignant U251 and A172 glioma cells resulted in cell proliferation inhibition, cell cycle arrest at the G0/G1 phase, and induction of cell apoptosis. Moreover, the downregulation of STAT3-dependent genes was demonstrated in these cells, i.e., *c-MYC*, *BCL-xL*, and *CCND1* [172]. The intratumoral administration of the STAT3 ODN-decoy in the xenograft glioma model based on U251 cells reduced tumor growth by inhibiting cancer cell proliferation and stimulating their apoptosis. The expression of *c-MYC*, *CCND1*, *CCNE1*, *BCL-2*, *BCL-xL*, and *BRIC5* decreased in STAT3-blocked tumors in reference to the controls [173]. 

In hepatocellular carcinoma, blocking STAT3 activity using the STAT3 ODN-decoy led to the inhibition of proliferation and cell cycle progression of HepG2, PLC/PRF/5, and H7402 cells [174]. In these cells, there was an induction of cell apoptosis, and the expression of apoptosis-related STAT3-dependent genes, such as *BCL-xL*, *CCND1*, and *c-MYC*, decreased [174]. Studies conducted on erlotinib-resistant and non-resistant SW480 colon cancer cells showed that the STAT3 ODN-decoy inhibited the growth of both cell lines [175]. The observed effect was associated with cell cycle arrest, cell proliferation inhibition, and cell apoptosis induction. Moreover, the STAT3 ODN-decoy-treated cells were characterized by reduced colony formation potential. The in vitro studies indicated that the blockage of STAT3 also inhibited SW480 cell migration ability. The effect on the reduction in the expression of the STAT3-dependent genes *BCL-xL* and *CCND1* was also demonstrated in vitro [175]. 

Sen et al. linked the ODN-decoy oligonucleotide strands using hexamethylene glycol spacers to increase oligonucleotide therapeutic stability, resulting in the generation of the cyclic STAT3 ODN-decoy [176]. The drug was applied systemically, which led to the growth inhibition of xenograft tumors made of UM-SCC1 cells in the head and neck squamous cell carcinoma (HNSCC) model. The therapeutic effect was connected with an inhibition of *BCL-xL* and *CCND1* expression in tumors [176].

**Table 1:** The oligonucleotide-based therapeutics targeting STAT3 delivered into cancer cells in a naked form. Legend: ↑—increase, ↓—decrease, nd—no data.

Oligo	Cancer	In Vitro Study		In Vivo Study		Ref.
		Cell Line	Effect	Cell Line/Route of Administration	Effect	
siRNA	Colorectal cancer	HCT-116 SW480	Apoptosis ↑	HCT-116 /intratumoral injection and electroporation	Tumor growth ↓	[135]
	Gastric cancer	SGC-7901	Cell cycle arrest	SGC-7901 /local electrotransfection	Tumor growth ↓, proliferation ↓	[136]
	Hepatocarcinoma	Bel-7402	Apoptosis ↑	nd	nd	[138]
	Lung cancer	Lewis lung cancer cell	Proliferation ↓ Apoptosis↑	nd	nd	[139]
		HCC827 HCC827ER H1975	Apoptosis ↑	nd	nd	[140]
	Leukemia	K562	Proliferation ↓ Apoptosis ↑ Cell cycle arrest	nd	nd	[141]
		SU-DHL-8 OCI-LY1	Proliferation ↓ Migration and invasion ↓	nd	nd	[142]
		nd	nd	MV4-11/intratumoral injection	Tumor growth ↓	[143]
	Myeloma	nd	nd	KMS-11/intratumoral injection	Tumor growth ↓	[143]
	Ovarian cancer	SKOV3 OVCAR3	Proliferation ↓ Apoptosis ↑	OVCAR3 /in vitro modified cells	Tumor growth ↓	[144]
		C13K SKOV3	Chemoresistance ↓	nd	nd	[145]
	Astrocytoma	A172 T98G	Viability ↓ Apoptosis ↑	nd	nd	[146]
	Retinoblastoma	ARPE-19 HRMECs Y79	Proliferation ↓	Y79 /intravitreal injection	Tumor growth ↓	[147]
	Oral squamous cell carcinoma	GFP-SAS HSC-4 HSC-3 KB	Proliferation ↓	nd	nd	[148]
	Breast cancer	MDA-MB-231	Apoptosis ↑	MDA-MB-231 /in vitro modified cells	Tumor growth ↓ Apoptosis ↑	[151]
		MDA-MB-231	Proliferation ↓ Migration ↓ Clonogenic survival ↓	nd	nd	[152]
		MDA-MB-231 HCC70	Invasion ↓	nd	nd	[82]
	Prostate cancer	DU145 LN-17	Proliferation ↓ Apoptosis ↑	nd	nd	[153]
		nd	nd	PC3 DU145 /intratumoral injection	Tumor growth ↓	[154]
shRNA	Ovarian cancer	CAOV3	Proliferation ↓ Apoptosis ↑	CAOV3/intratumoral injection /in vitro modified cells	Tumor growth ↓ Metastasis ↓	[155,156]
	Lung cancer	A549 SPC-A1 SK-MES-1	Viability ↓ Colony formation ↓ Chemoresistance ↓	A549 SK-MES-1 /in vitro modified cells	Radiosensitivity ↑	[157,158]
	Oral cancer	nd	nd	Hep-2 /intratumoral injection	Radiosensitivity ↑	[159]
	Head and neck cancer	HN5	Proliferation ↓	HN5 /in vitro modified cells	Tumor growth ↓	[160]
	Prostate cancer	PC-3M	Apoptosis ↑	PC-3M /intratumoral injection	Tumor growth ↓	[161]
	Breast cancer	MCF7-HER2	Invasiveness ↓ Tumorsphere formation ↓ Stemness ↓	nd	nd	[162]
	Hepatocellular carcinoma	HepG2.2.15	Proliferation ↓ Apoptosis ↑	HepG2.2.15/intratumoral injection	Tumor growth ↓	[163]
	Lymphoma	SCC-3	Proliferation ↓	SCC-3 /in vitro modified cells	Tumor growth ↓	[164]
ASO	Hepatocellular carcinoma	HCCLM3 SNU423 Huh7	Proliferation ↓ Migration ↓ Apoptosis ↑	HCCLM3/intraperitoneal injection	Angiogenesis ↓ Metastasis ↓	[165]
	Lymphoma	SUP-M2 KARPAS299	Proliferation ↓ Apoptosis ↑	A431 SUP-M2 /intravenous injection	Tumor growth ↓	[89]
ODN-decoy	Lung cancer	201T H1975	Apoptosis ↑ Colony formation ↓	201T H1975 /intravenous injection	Tumor growth ↓	[168]
	Breast cancer	MDA-MB-231	Proliferation ↓ Mammospheroid formation ↓ Apoptosis ↑ Chemoresistance ↓ Radiosensitivity ↑ Invasion ↓ Migration ↓	nd	nd	[92,95]
	Ovarian cancer	SKOV3 OVCAR3	Chemoresistance ↓ Proliferation ↓ Apoptosis ↑	SKOV3/intratumoral injection	Tumor growth ↓ Apoptosis ↑	[169,170,171]
	Glioma	U251 A172	Proliferation ↓ Apoptosis ↑	nd U251 /intratumoral injection	nd Tumor growth ↓ Apoptosis ↑	[172,173]
	Hepatocellular carcinoma	HepG2 PLC/PRF/5 H7402	Apoptosis ↑	nd	nd	[174]
	Colon cancer	SW480	Chemoresistance ↓ Migration ↓	nd	nd	[175]
	Head and neck cancer	nd	nd	UM-SCC1/intravenous injection	Tumor growth↓	[176]

## 5. shRNA-Based Therapeutics Targeting STAT3 Delivered into Cancer Cells by Viral-Based Carrier Systems

As mentioned above, shRNA can be delivered into the cells as a plasmid or viral vector. The most frequent method for plasmid delivery is electroporation or using a transfection agent. However, these methods are applicable for in vitro study or to modify cells in vitro for further in vivo experiments. Although, an intratumoral delivery of a plasmid-carrying shRNA sequence and subsequent local electroporation was also reported as an efficient method. However, using VLP as a vehicle that transfers genetic material into cells is considered one of the most effective methods allowing for stable expression of an shRNA construct. As VLPs belong to carrier-based drug delivery systems, the data related to the use of viral vectors are presented in a separate chapter. Moreover, the data describing the application of nucleic acid-based therapeutics delivered in the virus-based carrier to inhibit *STAT3* in cancer are summarized in Table 2.

Chen et al. found that *STAT3* shRNA reduces the ability of spheroid formation by EOC and SKOV3 ovarian cancer cells [177]. The Dickkopf-1 WNT signaling pathway inhibitor 1 (*DKK1*) was upregulated by the inhibition of miR-92a-1 in these *STAT3*-silenced ovarian cancer cells. In mice bearing *STAT3*-silenced ovarian SKOV3 tumors, treatment with paclitaxel reduced metastasis, inhibited tumor growth, and increased mice survival. The silencing of *STAT3* alone also inhibited tumor growth, but the effect was less than simultaneous treatment with *STAT3* shRNA and paclitaxel [177]. 

In choriocarcinoma, the silencing of *STAT3* by shRNA reduced the chemoresistance to methotrexate (MTX), fluorouracil (5-FU), and etoposide (VP16) of JEG-3 cells through the downregulation of nuclear factor interleukin 3 (*NFIL3*) [178].

STAT3 is an essential factor for oral cancer growth [179]. The silencing of *STAT3* by shRNA inhibited the proliferation of the SAS oral cancer cell line and reduced tumor growth in the xenograft oral cancer model [179].

The silencing of *STAT3* in 4T1 breast cancer cells by shRNA reduced *c-MYC* expression and TWIST protein level [180]. The activated SCARECROW (SCR) protein also decreased in these cells. After *STAT3* silencing, the invasive capacity of 4T1 cells was inhibited in vitro, and there was no metastasis in mouse-bearing *STAT3*-silenced 4T1 cells compared with non-modified cells. However, cell proliferation and cell cycle were not significantly different in 4T1 breast cancer cells after silencing *STAT3* compared with the control group. Tumor formation after the injection of 4T1/*STAT3*-silenced cells was inhibited in mice compared with the injection of control cancer cells [180]. 

Yang et al. silenced *STAT3* at mRNA and protein levels by shRNA in human SW1990 and 293T pancreatic cancer cells [181]. The silencing of *STAT3* suppresses these cells’ proliferation and invasion potentials. Moreover, the expression of *MMP2* and *VEGF* decreased after *STAT3* silencing compared with the control group in vitro [181]. Further analysis, using the same cell lines, was conducted in a xenograft model of pancreatic cancer. SW1990 cells, after shRNA-mediated silencing of *STAT3*, formed smaller tumors. Moreover, the expression of collagen IV, invasiveness abilities, and angiogenesis were also reduced in the *STAT3* shRNA-treated group. *STAT3*-silenced pancreatic tumors were characterized by the downregulation of the expression of STAT3-related genes such as *MMP7*, *MMP9*, and *IL-1β* [182].

*STAT3* silencing in HT-29 colorectal carcinoma cells resulted in their cell cycle arrest and the inhibition of proliferation. Moreover, it also reduced tumor growth and angiogenesis when tested in a xenograft colorectal cancer model. Although the *VEGFA* and *MMP2* expression decreased in *STAT3*-silenced colorectal tumors, there was no change in the expression of *FGF2* compared with the control group in vivo [183]. A high level of active STAT3 was found in colon cancer-initiating cells, which were also characterized by the expression of *ALDH* and *CD133* markers. ALDH+/CD133+, a subpopulation of SW480, HCT116, DLD-1, and HT-29 colon cancer cells, was treated with *STAT3* shRNA [184]. In vitro analyses after *STAT3* shRNA application showed that the expression of *CCND1*, *BRIC5*, *BCL-xL*, neurogenic locus notch homolog protein 1 (*NOTCH1*), and neurogenic locus notch homolog protein 3 (*NOTCH3*) decreased in treated cells. Further analyses showed that *STAT3* shRNA reduced the viability of colon cancer-initiating cells. Moreover, *STAT3* shRNA treatment also reduced tumor growth in vivo compared to the control group [184]. 

Combined therapy of the silencing of *STAT3* by shRNA with a Heat Shock Protein 90 (Hsp90) inhibitor SNX-2112 suppressed esophageal cancer stem-like cell (ECSLC) growth [185]. The simultaneous use of *STAT3* shRNA and SNX-2112 reduced ECSLC proliferation and colony formation capabilities in vitro. Moreover, this treatment decreased Multidrug Resistance-Associated protein 1 (*ABCB1*) and ATP-binding cassette superfamily G member 2 (*ABCG2*) gene expression in ECSLCs and caused cell cycle arrest. Researchers indicated that the combined treatment in vivo enhanced cancer cell apoptosis and led to tumor growth suppression compared with the SNX-2112 and sh*STAT3* group treated alone [185].

## 6. Oligonucleotide-Based Therapeutics Targeting STAT3 Delivered into Cancer Cells by Non-Viral-Based Carrier Systems

Nanoparticles can be functionalized with various molecules, potentially helping deliver therapeutic nucleic acids to cells. Nanoparticles and their functionalization can not only provide cargo protection against extracellular nucleases but also increase the pharmacokinetics and pharmacodynamics of therapeutics and increase their cellular specificity and their ability to penetrate cell membranes. Various non-viral carriers may increase the probability of the safe and efficient delivery of oligonucleotide therapeutics in vivo. The data describing the application of nucleic acid-based therapeutics delivered in the non-viral carrier to inhibit *STAT3* in cancer are summarized in Table 3.

### 6.1. siRNA-Based Therapeutics

Das and co-workers encapsulated the *STAT3* siRNA into carrier particles made of polyethyleneimine and poly (lactide-co-glycolide) [186]. The particles named NsiRNA were not sensitive to RNase treatment and efficiently entered the non-small-cell lung carcinoma A549 cells. The intraperitoneal injection of NsiRNA resulted in a significant STAT3 mRNA and protein reduction in the tumor samples in opposition to naked *STAT3* siRNA treatment, as indicated in a xenograft lung cancer model. Moreover, the therapeutic agent encapsulated in the carrier crossed the blood–brain barrier. NsiRNA induced cell apoptosis and arrested the cell cycle in the G0/G1 phase both in vitro and in vivo in contrast to naked *STAT3* siRNA. Moreover, the *STAT3* siRNA delivered by particles upregulated the expression of caspase-3 (*CASP3*) and caspase-9 (*CASP9*) and downregulated *CCND1*, *VEGF*, and *IL-6*, which was not the case after its application at the of naked form [186]. 

The application of *STAT3* siRNA encapsulated in micelles consisting of poly(d,l-lactic-*co*-glycolic acid) and chitosan was characterized by Zhao et al. in an ovarian cancer model [187]. An excellent cellular uptake of siRNA-loaded particles and effective *STAT3* silencing in SKOV3 ovarian cancer cells were observed. Moreover, the in vitro studies revealed that SKOV3 cells cured with siRNA/*STAT3*–PLGA/CSO micelles resulted in a higher inhibition of proliferation and induction of apoptosis compared with the control non-treated cells [187]. 

Zhang and co-workers analyzed a novel biodegradable siRNA delivery system based on cRGD-R9-PEG-PEICholesterol (rrPPC) conjugates [188]. RGD peptides can bind to αvβ3 integrins presented on the tumor microenvironment cells and thus are often used to enhance the specificity and efficacy of intracellular drug delivery [189]. The rrPPC nanoconjugates protected siRNAs from degradation by RNases. Moreover, rrPPCs were less cytotoxic than PEI25K, the “gold standard” for the transfection process. The rrPPC/si*STAT3* complexes were successfully internalized into C26 colon cancer cells, leading to *STAT3* silencing. C26 colon cancer cells treated with the complexes were characterized by reduced proliferation and increased apoptosis compared to the non-specific controls. Further in vivo research indicated that intraperitoneal injection of rrPPC/si*STAT3* complexes significantly inhibited C26 abdominal cavity metastasis. In turn, their intravenous injection reduced tumor burden, induced cancer cell apoptosis, and inhibited the angiogenesis process in the pulmonary metastases model [188]. 

Joshi et al. developed a method for the simultaneous delivery of *STAT3* siRNA and doxorubicin (DOX) to 4T1 mammary and CT26 colon carcinoma cells using pegylated chitosan lactate nanoparticles functionalized with TAT peptide and folate (siRNA/DOX-TAT-FACLP) [190]. TAT is a cell-penetrating peptide that can penetrate the cell membrane independent of receptor usage and temperature [191]. Folate (vitamin B) binds to cancer cells that frequently overexpress folate receptors. The in vitro application of doxorubicin or nanoparticles loaded with doxorubicin increased *STAT3* expression in 4T1 and CT26 cells. Moreover, applying nanoparticles carrying both *STAT3* siRNA and doxorubicin reduced *STAT3* expression in these cells. *STAT3* siRNA-containing nanoparticles and DOX-containing nanoparticles caused the cytotoxicity of breast and colorectal cancer cells. Still, the most significant cytotoxicity of these cells was caused by nanoparticles containing both oligonucleotide and chemotherapeutic agents. 4T1 and CT26 cells treated with siRNA/DOX-TAT-FACLP showed the highest level of apoptosis compared with other variants tested. Moreover, the application of particles loaded with both drugs was associated with a decrease in the level of BCL-2 protein and an increase in the BCL-2 interacting mediator of cell death (BIM) protein in 4T1 and CT26 cells. Further, in vivo analyses indicated that the injection of siRNA/DOX-TAT-FACLP into the tail vein inhibited cancer cell proliferation, migration, and invasion processes of breast and colon cancer cells. Additionally, it correlated with a decreased expression of *MMP2* and *MMP9* in tumor samples [190].

*STAT3* siRNA was delivered into 4T1 breast cancer cells using the pGensil-2 plasmid (pSi) [192]. After treatment, the level of STAT3 and STAT3-dependent proteins such as VEGF, MMP9, and MMP2 decreased in 4T1 cells. Moreover, the pSi-*STAT3*-treated 4T1 breast cancer cells indicated a reduced potential for induction angiogenesis and migration. Additionally, pSi-*STAT3s* embedded into cationic liposomes were used for in vivo study and applied by intravenous injection to mice bearing breast cancer metastases in the lungs. Tumor-bearing mice treated with pSi-*STAT3*/liposomes formed fewer metastases and smaller tumors than the control groups. Moreover, a decreased expression of *STAT3* and *VEGF* in neoplastic tissue was demonstrated. It was also shown that the in vivo administration of the pSi-*STAT3*/liposomes resulted in the induction of cancer cell apoptosis and the inhibition of the angiogenesis process in breast tumors [192]. 

PR39 is a porcine Cathelicidin rich in proline and arginine, and it is also involved in antimicrobial activities. Cathelicidin can efficiently penetrate the cell membranes and, therefore, has been used for the delivery of siRNA to cells [193]. PR39/*STAT3* siRNA complexes entered the 4T1 breast cancer cells, which resulted in the effective silencing of *STAT3*. The complexes did not affect the proliferation and cell cycle of 4T1 breast cancer cells but caused a higher inhibition of cell migration and invasion compared with the application of naked *STAT3* siRNA. Silencing *STAT3* using the PR39/*STAT3* siRNA complexes also decreased the expression of *MMP9* in breast cancer cells, which might explain the decrease in their migration potential [193]. 

Another research team developed a polyethyleneimine–polylactic acid–lipoic acid (PPL) micelle to simultaneously deliver *STAT3* siRNA and paclitaxel (PTX) into the breast cancer model [194]. The nanoparticles additionally have been coated with hyaluronic acid (HA) for better cellular uptake by CD44-overexpressed 4T1 breast cancer cells via an active targeting mechanism. 4T1 cells treated with HA/si*STAT3* PPL PTX particles were characterized by increased cell cycle arrest, reduced colony formation capacity, and increased apoptosis compared with cells treated with paclitaxel alone. Moreover, the in vitro application of the HA/si*STAT3*PPL particles reduced migration and the invasion of breast cancer cells. In vivo studies revealed that the intravenous administration of *STAT3* siRNA and paclitaxel-carrying particles to breast cancer-bearing mice significantly reduced tumor growth and metastasis compared to treating the mice with each agent separately. Furthermore, applying a carrier to deliver *STAT3* siRNA and paclitaxel did not cause side effects [194]. 

Other researchers simultaneously introduced *STAT3* siRNA and methotrexate into MCF7 breast cancer cells using mesoporous silica nanoparticles functionalized with chitosan (chMSNs) [195]. In vitro study indicated that nanoparticles loaded with *STAT3* siRNA resulted in more significant silencing of *STAT3* in MCF-7 cells than applying naked siRNA. Moreover, MTX/*STAT3*siRNA-loaded particles caused more significant toxicity toward MCF-7 cells than either free *STAT3* siRNA, methotrexate, or nanoparticles loaded with each agent alone [195]. 

In a study conducted by Ye et al., the therapeutic effect of 3′-cholesterol-modified *STAT3* siRNA (Chol-si*STAT3*) or Dicer substrate *STAT3* siRNA (Chol-Dsi*STAT3*) carried by particles made of the cationic diblock copolymer PLL[30]-PEG[5K] was investigated [196]. In a mouse model of breast cancer (based on 4T1 breast cancer cells), they showed that applying particles carrying Chol-si*STAT3* or Chol-Dsi*STAT3* led to tumor growth inhibition. However, particles loaded with Chol-Dsi*STAT3* had a more tremendous potential to reduce STAT3 on the protein level than Chol-si*STAT3* in 4T1 breast cancer in vivo [196]. 

Cationic liposomes containing *STAT3* siRNA and curcumin were administered to the B16F10 melanoma cells [197]. The application of curcumin/*STAT3* siRNA-loaded liposomes caused significant tumor growth inhibition in contrast with the treatment using control liposomes and naked *STAT3* siRNA in the melanoma cancer model. Moreover, the effectiveness of liposome penetration through the skin increased after using iontophoresis. Iontophoresis is a method that transports ionic therapeutic agents through the skin using low-level electric current. Among the tested therapy variants, the most significant reduction in melanoma growth in vivo was observed after the intratumoral administration of liposomes containing curcumin and *STAT3* siRNA and after using liposomes containing curcumin and *STAT3* siRNA with iontophoresis. Although tumor reduction was observed in both cases, the most considerable tumor reduction was observed with the application of drugs by intratumoral injection [197].

In a study conducted by Erdene-Ochir and co-workers, the nanoparticles based on alkylated cationic curdlan derivatives were used to treat melanoma [198]. The obtained particles were characterized by low cytotoxicity. The delivery of *STAT3* siRNA to B16 melanoma cells using these nanoparticles was very effective and caused a reduction in *STAT3* expression and an induction of cell apoptosis [198]. 

In another study, the delivery of *STAT3* siRNA into B16.F10 melanoma cells by polyplexes made of oleic acid- and stearic acid-modified polyethylenimine (PEI- p-*STAT3*-siRNA) decreased *STAT3* expression [124]. Further study revealed that the silencing of *STAT3* led to reduced *VEGF* expression and decreased cell viability [124]. 

Pan et al. developed a method for delivering siRNA deep into the skin via dissolving microneedles made of dextran 40, polyvinylpyrrolidone (PVP 17), and HA to treat melanoma [199]. However, to increase the oligonucleotide cellular uptake, they additionally used polyethyleneimine (PEI) to form complexes with siRNA. *STAT3* siRNA delivered by PEI vehicles effectively silenced *STAT3* and inhibited melanoma cell proliferation in contrast with the application of naked *STAT3* siRNA. Further in vivo study revealed that using microneedles that contained PEI carriers for *STAT3* siRNA delivery in a mouse melanoma model resulted in *STAT3* silencing and tumor growth inhibition [199]. 

### 6.2. shRNA-Based Therapeutics

Jiang and colleagues found that introducing *STAT3* shRNA into A2780CP and A2780 ovarian cancer cells inhibited *STAT3* gene expression [200]. After the in vitro transfection of *STAT3* shRNA carrying plasmid, the inhibition of cell proliferation and the induction of cell apoptosis were observed. This also led to a decrease in the BRIC5, BCL-2, and VEGF protein levels and an increase in cleaved caspase-3. However, for the research performed in vivo, they used the *STAT3* silencing plasmid enclosed in cationic liposomes to deliver the cargo to the tumor efficiently. Treatment with sh*STAT3*/lipoplexes reduced STAT3 protein levels and inhibited tumor growth. Further studies showed increased apoptosis in tumors treated with sh*STAT3*/lipoplexes and that cancer cell proliferation and angiogenesis were inhibited compared with the control [200]. 

The in vitro research using *STAT3* shRNA transfected into H1650 lung cancer cells showed that construct decreased STAT3 on the mRNA and protein levels [201]. The *STAT3* shRNA in vitro application resulted in the inhibition of cell proliferation and the induction of cell apoptosis. After the implantation of modified cancer cells in mice, tumor growth was significantly inhibited compared with the control. The authors also looked for a suitable carrier to help with in vivo *STAT3* shRNA delivery into the cancer cells. In a comparison study, they tested chitosan, vitamin E succinate–chitosan–histidine (VCH), and polyethyleneimine as potential carriers. Based on low toxicity, high integration into A549 lung cancer cells, and the silencing of *STAT3* expression as observed in vitro, they selected VCH particles for further research in cancer therapy [201]. 

### 6.3. ODN-Decoy-Based Therapeutics

Zhang et al. investigated methods for the efficient and safe delivery of the STAT3 ODN-decoy to esophageal squamous cell carcinoma [202]. First, they considered embedding the drug into liposomes, but due to the low transfection efficiency of liposomes, they developed another method of delivering the STAT3 ODN-decoy. The method was named ultrasound-targeted microbubbles combined with ultrasound. Ultrasound microbubbles were made of a SonoVue substance, a contrast agent used for ultrasound imaging. SonoVue is sulfur hexafluoride (SF6) surrounded by a phospholipid membrane, which forms microbubbles. Several experiments were used to study the efficiency of STAT3 ODN-decoy delivery into esophageal squamous cell carcinoma EC9706 cells, such as (i) ultrasonic microbubbles plus ultrasonic irradiation, (ii) liposomes plus ultrasonic irradiation, (iii) ultrasonic irradiation alone, and the application of (iv) ultrasonic microbubbles. The most effective STAT3 ODN-decoy delivery into EC9706 cells was obtained using ultrasonic microbubbles plus ultrasonic irradiation. It caused a higher percentage of apoptotic cells, the most significant inhibition of EC9706 cell proliferation, and the downregulation of *STAT3*, *BCL-xL*, and *CCND1*. Furthermore, in vivo studies indicated that STAT3 inactivation by the intravenous injection of ultrasound microbubble vesicles carrying the STAT3 ODN-decoy treated with ultrasound irradiation was the most effective in inhibiting esophageal squamous cell carcinoma growth. A similar effect was observed in the mouse group treated with the STAT3 ODN-decoy loaded in liposomes plus ultrasonic irradiation. Still, its efficiency was lower than in the mice that received the STAT3 ODN-decoy loaded ultrasonic microbubble plus ultrasonic irradiation [202]. 

Cationic solid lipid nanoparticles (SLNs) were an effective gene delivery system due to their high biocompatibility, stability, and low cytotoxicity [203]. Zhang et al. used SLN nanoparticles to introduce the STAT3 ODN-decoy into SKOV3 ovarian cancer. In vivo intratumoral administration of the SLN-STAT3 ODN-decoy into mice bearing SKOV3 tumors resulted in a significantly higher tumor growth inhibition compared with the treatment with non-specific scrambled ODN-decoy molecules. The SLN-STAT3 ODN-decoy induced ovarian cancer cell apoptosis, which was higher than the apoptosis of cancer cells after naked STAT3 ODN-decoy application. The observed effect was correlated with a decrease in pro-caspase-3, BCL-2, and survivin and an increase in caspase-3 and BAX protein levels. Moreover, the SLN-STAT3 ODN-decoy-treated mice contained a higher number of autophagic cells in the tumor tissues. Ovarian tumors in mice treated with the SLN-STAT3 ODN-decoy had reduced potential for invasion due to the downregulation of *VEGF*, *MMP9*, *CDH2*, and Vimentin (*VIM*) and elevated levels of *CDH1*. The study showed no toxic side effects caused by the SLN-STAT3 ODN-decoy in mice [203]. 

The effect of the combinatory therapy based on trastuzumab (TRAZ) with the STAT3 ODN-decoy delivered by nanoparticles in breast cancer that overexpressed HER2 was investigated [204]. Trastuzumab is the antibody that targets the HER2 receptor and is used to treat HER2-positive cancers. The asymmetric hybrid lipid/polymer particles were formed from calcium phosphate as the solid kernel coated with hyaluronic acid (CaP@HA). The STAT3 ODN-decoy delivered with trastuzumab by CaP@HA reduced BT474R breast cancer cells’ resistance to trastuzumab. Moreover, the application of CaP@HA for the delivery of the STAT3 ODN-decoy significantly increased the oligonucleotide drug’s cellular uptake and serum stability. The BT474R cells’ viability decreased, and apoptosis increased drastically after the administration of TRAZ with the STAT3 ODN-decoy packed into CaP@HA as opposed to the application of TRAZ alone or in combination with a naked STAT3 ODN-decoy. Moreover, the use of a vehicle for drug co-delivery significantly decreased the expression of *BCL-2*, *MCL-1*, *BRIC5*, and mucin 4 (*MUC4*) compared with the simultaneous TRAZ and STAT3 ODN-decoy application without practice. In the mouse breast cancer xenograft model, the treatment of CaP@HA loaded with the STAT3 ODN-decoy plus TRAZ inhibited tumor growth and induced tumor cell apoptosis to a greater extent than the application of a naked oligonucleotide therapeutic in combination with TRAZ or TRAZ alone. Moreover, mice treated with CaP@HA loaded with the STAT3 ODN-decoy and TRAZ had a higher survival rate than the other treated groups [204]. 

Zhang et al. conducted studies on the delivery of the STAT3 ODN-decoy (STAT3d) using gold nanoparticles (AuNPs) [133]. The constructed particles might act in two manners due to blocking STAT3 molecules and possibly using gold nanoparticles for radiosensitization. An aptamer that recognizes nucleolin (NUAP) overexpressed in head and neck cancer cells was also added to AuNP. AuNP-NUAP-STAT3d sensitized FaDu head and neck cancer cells to radiotherapy and inhibited cancer cell proliferation in vitro. Combining radiotherapy with anti-EGFR antibodies is recommended for treating head and neck cancer. However, applying the obtained AuNP-NUAP-STAT3d particles showed a more substantial sensitization effect of radiotherapy than the humanized anti-EGFR antibody (Cetuximab) [133].

**Table 3:** The oligonucleotide-based therapeutics targeting STAT3 delivered into cancer cells by non-viral-based carrier systems. Legend: ↑—increase, ↓—decrease, nd—no data.

Oligo	Cancer	In Vitro Study			In Vivo Study			Ref.
		Carrier	Cell Line	Effect	Carrier	Cell Line /Route of Administration	Effect	
**siRNA**	Lung cancer	PEI/PLGA nanoparticles	A549	Apoptosis ↑ Cell cycle arrest	PEI/PLGA nanoparticles	A549/intraperitoneal cavity injection	Apoptosis ↑ Cell cycle arrest	[186]
	Ovarian cancer	PLGA/CSO micelles	SKOV3	Proliferation ↓ Apoptosis ↑	nd	nd	nd	[187]
	Colon cancer	rrPPC nanoparticles	C26	Proliferation ↓ Apoptosis ↑	rrPPC nanoparticles	C26/intravenous injection	Metastasis ↓ Tumor growth↓ Apoptosis ↑ Angiogenesis ↓	[188]
	Colon cancer and breast cancer	TAT-FA-CLP nanoparticles	CT26 4T1	Chemoresistance ↓ Apoptosis ↑	TAT-FA-CLP nanoparticles	CT26 4T1 /tail vein injection	Proliferation ↓ Migration ↓ Invasion ↓	[190]
	Breast cancer		4T1	Angiogenesis↓ Migration ↓	Liposomes	4T1 /tail vein injection	Metastasis ↓ Tumor growth↓ Apoptosis ↑ Angiogenesis ↓	[192]
		PR39 complexes	4T1	Migration ↓ Invasion ↓	nd	nd	nd	[193]
		HA/PPL micelles	4T1	Chemoresistance↓	HA/PPL micelles	4T1 /intravenous injection	Metastasis ↓ Tumor growth↓	[194]
		chMSNs	MCF7	Chemoresistance↓	nd	nd	nd	[195]
		nd	nd	nd	PLL-PEG nanoparticles	4T1	Tumor growth↓	[196]
	Melanoma	nd	nd	nd	Cationic liposomes	B16F10/intratumoral injection	Tumor growth ↓	[197]
		Cationic curdlan nanoparticles	B16	Apoptosis ↑	nd	nd	nd	[198]
		PEI polyplexes	B16.F10	Cell viability ↓	nd	nd	nd	[124]
		PEI complexes + microneedles	B16.F10	Proliferation ↓	PEI complexes + microneedles	B16.F10/intratumoral injection	Tumor growth↓	[199]
**shRNA**	Ovarian cancer	nd	A2780CPA2780s	Proliferation ↓ Apoptosis ↑	Cationic liposomes	A2780CP/intraperitoneal cavity injection	Tumor growth↓ Apoptosis ↑	[200]
	Lung cancer	nd	H1650	Proliferation ↓ Apoptosis ↑	VCH nanoparticles	H1650 /in vitro modified cells	Tumor growth↓	[201]
**ODN-decoy**	Esophageal squamous cell carcinoma	Ultrasound SonoVue microbubbles + irradiation	EC9706	Proliferation ↓	Ultrasound SonoVue microbubbles + irradiation	EC9706 /intravenous injection	Tumor growth↓	[202]
	Ovarian cancer	nd	nd	nd	SLN nanoparticles	SKOV3/intratumoral injection	Tumor growth↓ Apoptosis ↑	[203]
	Breast cancer	CaP@HA nanoparticles	BT474R	Chemoresistance↓ Cell viability ↓ Apoptosis ↑	CaP@HA nanoparticles	BT474R/intravenous injection	Tumor growth↓ Apoptosis ↑ Survival ↑	[204]
	Head and neck cancer	AuNP-NUAP nanoparticles	FaDu	Radiosensitivity ↑ Proliferation ↓	nd	nd	nd	[133]

## 7. Oligonucleotide-Based Therapeutics Targeting STAT3 in Cancer Investigated in the Clinic

As indicated above, numerous in vitro and in vivo assays testing oligonucleotide-based agents that either decrease STAT3 expression or directly inhibit STAT3 DNA-binding ability were reported. These reports have shown encouraging results in preclinical models far beyond proof-of-concept studies. However, only a few nucleic acid-based approaches have advanced human clinical testing. Therefore, optimizing these nucleotide therapeutics’ specificity, potency, stability, and delivery is essential for enhancing their therapeutic benefits in the clinic. In Table 4 and below, the current reports on applying anti-STAT3 oligonucleotide-based therapeutics in clinics are summarized. 

### 7.1. siRNA-Based Therapeutics

The only ongoing clinical trial using *STAT3* siRNA is currently recruiting patients [205]. The aim of this phase I study is to identify the optimal dose and adverse events (AEs) of CpG-*STAT3* siRNA CAS3/SS3 (CAS3/SS3) in combination with localized radiation therapy in the treatment of patients with relapsed or refractory NHL (NCT04995536). CAS3/SS3 is a molecule comprising a CpG oligonucleotide and a *STAT3* siRNA that selectively targets the TLR9 receptor and *STAT3* mRNA, respectively. They act together to interfere with the cancer cells’ growth ability. Radiotherapy utilizes high-energy X-rays to kill cancer cells and reduce the tumor’s volume. Accordingly, the administration of CAS3/SS3 combined with local radiotherapy may increase the number of inactivated tumor cells [205]. Eighteen patients were enrolled in the trial starting in 2022. Parameters including the incidence of adverse events, dose-limiting toxicity, overall disease response, response duration, the suppression of *STAT3* expression and STAT3 activation of downstream targets, and local or systemic immune responses are to be measured in the study. The estimated completion date of the study is 2024 [205].

### 7.2. ASO-Based Therapeutics

AZD9150 (Danvatirsen) is an antisense oligonucleotide (ASO) inhibitor of STAT3 that has shown clinical activity in several phase I/II clinical studies. Up to January 2023, thirteen studies on AZD9150 in cancer treatment were recorded in the ClinicalTrials.gov database [206]. Among them are six completed, six active, and one terminated clinical trial. Clinical trials include both monotherapies and combined therapies. Combinatory therapies compromise using *STAT3* ASO simultaneously with radiotherapy, chemotherapy, or immunotherapy. 

One of the first-in-human studies was a phase I/II clinical trial of the next-generation antisense oligonucleotide inhibitor of STAT3 (ISIS 481464, IONIS-STAT3Rx). The trial was carried out in patients with solid and hematologic malignancies refractory to at least one prior systemic therapy (NCT01563302). Of the thirty patients enrolled in the study, including twenty-seven patients with diffuse large B cell lymphoma (DLBCL), ten received a dose of 2 mg/kg and twenty received a dose of 3 mg/kg. Both tested AZD9150 doses proved to be safe and well tolerated. In DLBCL patients, two partial and two complete responses with a median duration of response of 10.7 months occurred after AZD9150 administration at both tested dose levels [207]. As no significant difference in patients’ progression-free and overall survival between the 2 mg/kg and 3 mg/kg dose levels was observed, the higher dose was recommended for phase II. Common mild AEs occurred among AZD9150-treated patients in this trial, including transaminitis, fatigue, and thrombocytopenia. In general, AZD9150 was safe and effective in a subset of patients with DLBCL heavily pretreated with systemic therapy. However, administration with AZD9150 influenced the selected immune cell populations, as indicated in the peripheral blood analyses performed in this study [207]. 

AZD9150, given in combination with Acalabrutinib (kinase inhibitor), was analyzed in a phase Ib trial conducted in 2018–2021, focusing on treating hematological malignancies (NCT03527147). The study was carried out in thirty patients with relapsed or refractory aggressive non-Hodgkin’s lymphoma (NHL), and DLBCL reported no safety concerns about the proposed combination [208].

Other studies on AZD9150 in combination with checkpoint immunotherapies were also conducted. A phase I/II trial of AZD9150 in combination with durvalumab (anti-PD-L1 antibody) in Japanese patients with advanced solid tumors (NCT03394144) was conducted in the years 2018–2019 [209]. Eleven people aged ≥20 years with histologically confirmed solid cancers refractory to systemic therapy were recruited and randomly assigned to two cohorts. The first group included AZD9150 monotherapy; in the second group, patients received AZD9150 in combination with durvalumab. In the second cohort, abnormal liver function, decreased neutrophil counts, and decreased platelet counts were observed, requiring a reduction in the AZD9150 dose. Except for those mentioned, one case of eosinophilia requiring dose reduction was reported. In 90.9% of patients, mild Aes, such as decreased platelet count and increased ALT/AST/γGT, were observed. Generally, both AZD9150 monotherapy and combinatorial therapy with durvalumab demonstrated a good safety profile in Japanese patients with advanced solid malignancies [210]. 

Another phase Ib multicenter trial tested durvalumab as a monotherapy and combined with AZD9150 in patients with relapsed or refractory DLBCL (NCT02549651). The combined treatment was generally safe in the group of patients with relapsed or refractory DLBCL; however, limited antitumor activity was indicated [211].

Attempts to treat early-stage non-small-cell lung cancer (NSCLC) with AZD9150 in combination with durvalumab were undertaken in 2019 as a phase II randomized trial (NCT03794544). The study reported the safety and clinical activity of the combined formulation. Outcome measures, such as tumor and microbiome biomarkers, as well as mRNA signatures in blood samples, including basic tumor *PD-L1* and *CD73* gene expression levels, were investigated. According to transcriptomic analysis, the durvalumab plus AZD9150-treated group did not reveal any changes related to immune cell function in peripheral blood. Therefore, further testing of these drugs in the model of resectable NSCLC is warranted [212,213]. 

Moreover, five active, non-recruiting clinical trials concerning using AZD9150 for cancer treatment were found [206]. One of them, a phase Ib/II multicentre trial, investigates the safety profile, pharmacokinetic behavior, and initial antitumor efficacy of AZD9150 formulation combined with durvalumab in patients with relapsed metastatic head and neck squamous cell carcinoma (HNSCC) (NCT02499328) [214]. The study started in 2015. The study is designed as a two-part study consisting of a dose escalation and dose expansion. The first part will establish the maximum tolerated doses (MTDs) for each tested agent, with an observation focused on the occurrence of adverse events. Dose escalation will be performed in a group with both recurrent and metastatic (RM) HNSCC. The study will enroll 68 to 266 eligible patients who will be randomly divided into one of the arms or non-randomized arms. The planned completion date is 2023 [214]. 

In 2018, a phase Ib/II clinical trial was launched in the USA (NCT03421353). The safety, tolerability, pharmacokinetics, and preliminary antitumor activity of AZD9150 and durvalumab, with or without chemotherapy, will be analyzed in this study [215]. Seventy-six patients were enrolled in this clinical trial. This multicenter study is conducted in two parts. Firstly, the study will assess the safety of combinatorial treatment with durvalumab plus AZD9150 alone or in combination with selected chemotherapy regimens. In this part, the patients with advanced solid tumors resistant to standard chemotherapy will receive the treatment. Secondly, the method of AZD9150 administration will be compared to determine the bioavailability of the formulation following subcutaneous (SC) and intravenous (IV) injection. In the second part of the study, fifty-five to sixty-two volunteers will be recruited and randomly assigned to groups receiving AZD9150, either SC or IV. The estimated study completion date is the end of 2025 [215].

According to ClinicalTrials.gov, only one clinical trial testing the use of STAT3 ASO is recruiting. It is a multicenter phase II trial conducted in patients suffering from metastatic NSCLC (NCT03334617). This study has a modular structure, which allows for a preliminary assessment of the effectiveness, safety, and tolerability of various treatment formulations, including durvalumab, in combination with AZD9150. Recruitment for this study began in 2017. The study is mainly aimed at patients who have progressed on an anti-PD-1/PD-L1 therapy [216].

Among terminated studies, one item can be found. A study registered under the number NCT02417753 aimed to measure changes in immunological parameters in the malignant ascites in patients with advanced cancer after AZD9150 treatment. The study was closed because of the inability to find eligible patients [217].

### 7.3. ODN-Decoy-Based Therapeutics

The first and only study of an ODN-decoy targeting STAT3 in humans was registered in 2008 as an early phase I trial (NCT00696176) and completed in 2011 [218]. In this study, the STAT3 ODN-decoy was injected intratumorally to measure the inhibition of STAT3 target gene expression, STAT3 activation level, and apoptosis in head and neck tumors. Thirty-two patients participated in the study. None of the patients suffered from side effects. A kinetic study in a xenograft model of HNSCC was carried out to support the proposed study design. The results demonstrated that the administration of the STAT3 ODN-decoy decreased the expression of STAT3 target genes [176].

## 8. Conclusions—Drug Carriers Matter 

Inhibition of STAT3 activity in cancer is beneficial, as numerous reports indicate [219,220,221,222]. Various molecules can be used to target the STAT3 signaling pathway. Molecules that block ligands and receptors of the STAT3 signaling pathway, inhibitors of upstream tyrosine kinases, molecules that activate a negative feedback loop, and finally, inhibitors interacting directly with STAT3 were tested in many studies. Although the application of these substances resulted in beneficial outcomes, and some even were/are tested in clinics, they have several limitations. Among others, the most crucial is the existence of many upstream proteins that can activate STAT3, which results in compensating effects after blocking one particular target. Additionally, some of these molecules’ relatively low specificity is another issue. Thus, oligonucleotide therapeutics can constitute an exciting alternative. The main advantage of inhibiting STAT3 with nucleic acid-based therapeutics is their high specificity based on the complementarity of the therapeutics sequence with the target sequence. Conversely, oligonucleotide-based therapeutics struggle with stability, toxicity, sensitivity to nucleases, and specificity toward cell type, which hampers their applicability. Among the various solutions proposed to overcome these problems (including the use of advanced chemistry), embedding the oligonucleotide in the carrier emerges as an interesting strategy. The review summarizes data on nucleic acid-based therapeutics targeting STAT3 in cancer treatment, indicating beneficial therapeutic effects with both naked and carrier-embedded oligonucleotides. Few studies have directly compared the use of oligonucleotide drugs in both formulations. However, some studies showed that STAT3-targeting oligonucleotides transported in particles provided a significantly more favorable therapeutic effect than particles delivered naked at the same dose [186,204]. However, particles loaded with anti-STAT3 oligonucleotide therapeutics have yet to be tested in clinical trials.

## 9. Perspectives

Carriers not only can protect the oligo from nucleases and safely deliver it into cells but also, upon functionalization, selectively target cancer cells or other cells within the TME. According to the basic concept of using nanoparticles in cancer treatment, their accumulation in the cancer tissue occurs due to the effect of increased permeability and retention (EPR). However, the passive accumulation of nanoparticles in the TME is rather limited since only approximately 0.7% of the administered nanoparticle doses reached the tumor [223]. In addition, cargo released into the tumor microenvironment may be degraded/inactivated before reaching the target. For this reason, the active transport that delivers drugs selectively to a defined cell type in the TME can be more beneficial for therapy. Active targeting can be accomplished using ligands that recognize cells specifically. The application of functionalized particles to actively deliver drugs to cancer cells surpasses the effectiveness of particles without functionalization [224]. In addition, due to the higher efficiency, the administered dose of the particles may be reduced, which may be beneficial in terms of potential side effects. The advantages offered by carriers are of particular importance for oligonucleotide therapeutics delivery. Firstly, combining a high specificity of therapeutics toward their targets and nanoparticles toward cell type can generate very efficient formulation. Moreover, particle-mediated delivery can prolong nucleic acid-based molecules’ intercellular presence and activity [225]. As was pointed out above, the STAT3-targeting oligonucleotide therapeutics transported in particles provided a much more beneficial therapeutic effect than molecules delivered in the naked form at the same dose [186,204].

This report is focused on applying oligonucleotide therapeutics that directly target cancer cells. However, one should be aware that silencing STAT3 activity could also alter the functions of non-tumor cells within the TME. Due to the biology of STAT3 and its role in cancer development, therapies targeting this molecule in immune cells, cancer-associated fibroblast, or endothelial cells are also under investigation. Moreover, in vivo studies of STAT3 inhibition, independent of whether the drugs (including oligonucleotide-based ones) are delivered in naked or embedded form, target various cells within the TME unless active targeting is used. Additionally, the complexity of the problem increases due to the biology of STAT3. As a result of alternative splicing, two STAT3 isoforms are produced, STAT3α and STAT3β. Both isoforms perform different functions in physiological and pathological conditions. Moreover, STAT3 can be activated by both canonical and non-canonical pathways, resulting in various post-translational modifications that affect its activity. Since oligonucleotide-based therapeutics can not only exert therapeutic effects by inhibiting their target at the nucleic acid and protein levels but also by editing genes, their application to control the STAT3 structure is possible. Perhaps therapies based on selecting the most desirable variant of STAT3 showing therapeutic properties rather than blocking its activity are an interesting approach. The use of oligonucleotide therapeutics makes it possible to achieve such a goal. Combining a specific drug with active delivery in a carrier could increase the outcome and safety of such therapy.

## Figures and Tables

**Table 2:** The oligonucleotide-based therapeutics targeting STAT3 delivered into cancer cells by viral-based carrier systems. Legend: ↑—increase, ↓—decrease, nd—no data.

Oligo	Cancer	In Vitro Study		In Vivo Study		Ref.
		Cell Line /Type of Viral Vector	Effect	Cell Line /Route of Viral Vector Administration	Effect	
shRNA	Ovarian cancer	SKOV3 /Lentiviral vector	Spheroid formation ↓	SKOV3 /in vitro modified cells	Metastasis ↓ Tumor growth ↓	[177]
	Choriocarcinoma	JEG-3 /Lentiviral vector	Chemoresistance ↓	nd	nd	[178]
	Oral cancer	SAS /Lentiviral vector	Proliferation ↓	SAS /in vitro modified cells	Tumor growth ↓	[179]
	Breast cancer	4T1 /Lentiviral vector	Invasiveness ↓	4T1 /in vitro modified cells	Metastasis ↓ Tumor growth ↓	[180]
	Pancreatic cancer	SW1990 /Lentiviral vector	Proliferation ↓ Invasion potential ↓	SW1990 /in vitro modified cells	Tumor growth ↓ Angiogenesis ↓	[181,182]
	Colorectal carcinoma	HT-29 /Lentiviral vector	Proliferation ↓	HT-29 /in vitro modified cells	Tumor growth ↓ Angiogenesis ↓	[183]
	Colon cancer	SW480 HCT116 /Lentiviral vector	Viability ↓	HCT116 /in vitro modified cells	Tumor growth ↓	[184]
	Esophageal cancer	Eca109 HEEC /Lentiviral vector	Proliferation ↓ Colony formation ↓ Chemoresistance ↓ Cell cycle arrest	Eca109 HEEC /in vitro modified cells	Apoptosis ↑ Tumor growth ↓ Chemoresistance ↓	[185]

**Table 4:** The oligonucleotide-based therapeutics targeting STAT3 in cancer investigated in the clinic.

Oligo	Status	Phase	Start Date	Completion Date	Disease	Enrollment	Study Identifier	Sponsor/Collaborator	Location Countries	Combination
*ASO* AZD9150	Completed	I/II	27 February 2012	23 March 2016	DLBCL Lymphoma Advanced cancers	64	NCT01563302	Ionis Pharmaceuticals Inc. AstraZeneca	USA	None
	Completed	I/Ib	May 2013	February 2015	Advanced and metastatic HCC	58	NCT01839604	AstraZeneca Ionis Pharmaceuticals Inc.	Hong Kong, Japan, South Korea, Taiwan	None
	Terminated (due to inability to find eligible patients)	II	3 April 2015	7 April 2016	Ovarian cancer Gastrointestinal cancer ascites	1	NCT02417753	National Cancer Institute (NCI)	USA	None
	Active, not recruiting	Ib/II	6 August 2015	29 December 2023	HNSCC Advanced solid tumors	340	NCT02499328	AstraZeneca MedImmune LLC	USA, Belgium, Germany, Italy, Spain, UK	Durvalumab
	Completed	Ib	13 July 2016	4 February 2019	DLBCL	32	NCT02549651	MedImmune LLC	USA, France, Ireland, UK	Durvalumab
	Active, not recruiting	I	3 October 2016	31 March 2023	Muscle invasive Bladder cancer	156	NCT02546661	AstraZeneca	USA, Canada, France, Spain, UK	Durvalumab
	Active, not recruiting	II	2 March 2017	31 March 2023	Pancreatic cancer Colorectal cancer NSCLC	53	NCT02983578	M.D. Anderson Cancer Center National Cancer Institute (NCI) AstraZeneca	USA	Durvalumab
	Recruiting	II	18 December 2017	2 January 2026	Metastatic NSCLC	530	NCT03334617	AstraZeneca	USA, Austria, Canada, France, Germany, Israel, South Korea, Spain	Durvalumab
	Completed	I	30 January 2018	12 April 2019	Advanced solid malignancies	11	NCT03394144	AstraZeneca	Japan	Durvalumab
	Active, not recruiting	Ib/II	7 February 2018	31 December 2025	NSCLC Advanced solid tumors	76	NCT03421353	AstraZeneca	USA	Durvalumab Chemotherapy: Cisplatin/ 5-Flourouracil/ Carboplatin/ Gemcitabine/ Nab-paclitaxel
	Completed	Ib	19 June 2018	31 March 2021	NHL DLBCL	30	NCT03527147	Acerta Pharma BV AstraZeneca	USA	Acalabrutinib
	Active, not recruiting	Ib	27 December 2018	26 March 2026	Metastatic NSCLC	258	NCT03819465	AstraZeneca	USA, Austria, Belgium, Canada, Korea, Poland Spain, Russia, Taiwan, Thailand	Durvalumab Chemotherapy: Pemetrexed/ Carboplatin/ Gemcitabine/ Cisplatin/ Nab-paclitaxel
	Completed	II	8 March 2019	13 January 2021	Early-stage NSCLC	84	NCT03794544	MedImmune LLC	USA, Canada, France, Italy, Portugal, Spain, Switzerland	Durvalumab
*ODN-decoy*	Completed	0	August 2008	August 2011	Head and neck cancer	32	NCT00696176	University of Pittsburgh	USA	None
*CpG-siRNA*	Recruiting	I	1 August 2022	27 January 2024	Lymphoma	18	NCT04995536	City of Hope Medical Center National Cancer Institute (NCI)	USA	Radiation therapy
